# Gut microbiome and short-chain fatty acids associated with the efficacy of growth hormone treatment in children with short stature

**DOI:** 10.3389/fped.2025.1557878

**Published:** 2025-03-31

**Authors:** Pingsihua He, Yongfen Lyu, Xinyuan Shen, Wenxin Liu, Ying Zhang, Yan Li, Yuying Huang, Liya Xu, Liang Zhang, Sheng Guo

**Affiliations:** ^1^ Department of Endocrine and Metabolism, Shanghai Children’s Hospital, School of Medicine, Shanghai Jiao Tong University, Shanghai, China; ^2^ Department of Traditional Chinese Medicine, Shanghai Children’s Hospital, School of Medicine, Shanghai Jiao Tong University, Shanghai, China

**Keywords:** microbiota, SCFAs (short-chain fatty acids), growth hormone treatment, short stature, IGF-1 (insulin-like growth factor 1)

## Abstract

**Objective:**

To investigate associations between fecal microbiota, short-chain fatty acids (SCFAs), and the efficacy of recombinant human growth hormone (rhGH) treatment in children with growth hormone deficiency (GHD) or idiopathic short stature (ISS).

**Methods:**

A 2-phase cohort study was conducted. Phase I included 102 participants (GHD: *n* = 33, ISS: *n* = 28, controls: *n* = 41) for cross-sectional analysis using 16S rRNA sequencing and targeted metabolomics to compare microbial diversity, predicted metabolic pathways, and SCFA levels. Phase II longitudinally monitored 61 rhGH-treated children (GHD = 33, ISS = 28) over 2 years, assessing growth velocity, IGF-1 levels, and fecal microbiota/SCFA dynamics. Statistical analyses included alpha/beta diversity metrics, LEfSe, PERMANOVA, and redundancy analysis (RDA) to link microbial/SCFA profiles with clinical outcomes.

**Results:**

(1). Microbiota Dysbiosis: Untreated GHD/ISS children exhibited reduced beneficial taxa (e.g., *Faecalibacterium*, *Akkermansia*) and increased pathobionts (e.g., *Streptococcus*, *Collinsella*) compared to controls (PERMANOVA: *R*^2^ = 0.114, *P* = 0.001). (2). Metabolic Pathways: GHD/ISS groups showed enrichment in xenobiotic degradation (e.g., atrazine) and deficits in nutrient-associated pathways (e.g., carotenoid biosynthesis). (3). rhGH Effects: Treatment increased beneficial taxa (e.g., *Bifidobacterium*, *Faecalibacterium*) and modulated amino acid/lipid metabolism pathways (e.g., glycine-serine-threonine metabolism, *P* = 0.035). (4). SCFAs and Growth Velocity: Higher growth velocity percentiles correlated with elevated acetic acid (GHD-treated: 1952 ± 962.4 vs. untreated: 1290 ± 886.0 μg/g, *P* = 0.037) and butyric acid levels.

**Conclusion:**

GHD, ISS, and healthy children have different fecal microbiota compositions and SCFA metabolisms. rhGH therapy partially restores microbial balance and alters metabolic pathways, with SCFA levels associated with treatment efficacy. These findings highlight the gut microbiome as a potential modulator of rhGH response and provide insight into microbiota-targeted therapies to improve growth outcomes (e.g., “probiotic interventions”).

## 1 Introduction

Short stature is generally defined as a condition where a person's height falls below the −2.0 standard deviation score (SDS) for their age and gender in a given population (1). This condition affects approximately 2.5% of children and is one of the most common reasons for them to consult a pediatric growth specialist (2). Childhood short stature can be an early sign of chronic illness or psychological deprivation, or it may simply be a common variant. A wide range of conditions can affect growth, and the European Society of Pediatric Endocrinology has identified over 100 main, secondary, and idiopathic diseases that can lead to short stature (3). Among these, familial short stature and constitutional delayed growth and puberty (CDGP) are the most common causes.

In addition to the physical problems it can cause, untreated short stature often leads to significant anxiety in children and their caregivers. At school, children with short stature may suffer from low self-esteem, anxiety, and even bullying (4, 5). However, in the case of the two non-pathological development patterns mentioned above, there is no need for excessive worry or action. However, when short stature is caused by other underlying or secondary factors, it should be treated properly (6). Early identification of children with short stature and the subsequent provision of appropriate interventions and treatments are of paramount importance (7). According to the International Classification of Paediatric Endocrine Diagnoses, three primary categories of etiology have been identified for short stature. The first category encompasses primary growth disorders, which include genetic syndromes, small for gestational age with failure of catch-up growth, and skeletal dysplasias. The second category comprises secondary growth disorders, which are caused by hormonal, nutritional, or environmental factors, or by specific organ diseases. The third category is idiopathic short stature (ISS) (8). In the past 35 years, growth hormone (GH) has emerged as a pivotal treatment for pathological short stature in children. The use of pediatric recombinant human growth hormone (rhGH) has progressed from its initial application to address growth hormone deficiency (GHD) to its current use to augment height in conditions where the body's own GH production is adequate (9). The present indications for rhGH treatment include conditions such as short stature due to Turner syndrome, Prader-Willi syndrome, chronic renal insufficiency, GH insufficiency/deficiency, small for gestational age, and ISS. In adults, the recognized medical uses of rhGH include, but are not limited to, the treatment of the wasting syndrome of HIV/AIDS and GH deficiency (10).

rhGH is a protein that is produced to be nearly identical to the primary form of naturally occurring human growth hormone. This hormone plays a crucial role in promoting tissue growth, linear growth, as well as the metabolism of protein, carbohydrate, lipid, and mineral (11). rhGH stimulates growth through two main mechanisms. First, it activates the MAPK/ERK pathway by binding to receptors on target cells, which directly stimulates the division and proliferation of cartilage cells. Second, it stimulates the production of insulin-like growth factor 1 (IGF-1) via the JAK-STAT signaling pathway, thereby promoting the activity of osteoblasts and cartilage cells and stimulating bone growth (12). IGF-1, a growth factor released by the liver, has been shown to promote longitudinal bone development through both endocrine and paracrine/autocrine actions. Recent studies (13) on germ-free mice have highlighted the significant regulatory role of short-chain fatty acids (SCFAs) produced by intestinal microbiota in the generation of IGF-1, in addition to the role of growth hormone. Research findings (14, 15) have indicated that levels of IGF-1 were notably elevated in colonized mice 1 and 8 months following colonization with gut microbiota in comparison to germ-free mice. Prolonged gut microbial colonization has been observed to stimulate both longitudinal and radial bone development in adult mice. Moreover, studies have revealed that children with short stature exhibit a distinct gut microbiota composition compared to their normal counterparts, resulting in microbiota dysbiosis. Probiotic or prebiotic supplementation has been demonstrated to rectify these imbalances and enhance growth in malnourished children. In summary, the existing evidence (16) suggests a close relationship between the GH-IGF1 axis and the intestinal microbiota, and the interaction between the GH-IGF1 axis and the gut microbiome plays a significant role in individual growth and development.

While emerging evidence (17–19) suggests that gut microbiome-host crosstalk plays a role in growth regulation, critical mechanistic gaps remain regarding microbiota-derived SCFA signaling in pediatric short stature and its therapeutic implications for rhGH responsiveness. The current evidence (20, 21) is deficient in its extensive profiling of microbial communities across etiological subtypes (GHD vs. ISS) and is inadequate in demonstrating causal relationships between microbial metabolic output and rhGH treatment trajectories. Furthermore, although some studies have linked microbiota-SCFA to serum IGF levels, the impact of the microbiota-SCFA profile on the efficacy of long-term rhGH treatment remains uncertain (22), which limits translational applications. To fill these knowledge gaps, we devised a two-phase exploratory cohort study that investigated two key questions: whether unique gut microbiota/SCFA profiles distinguish between GHD, ISS, and healthy controls. Determine if baseline or treatment-induced microbial characteristics predict growth velocity and IGF-1 normalization. Phase I employs multi-omics integration (16S rRNA sequencing, targeted metabolomics) to compare taxonomic and functional profiles across 102 participants (GHD = 33, ISS = 28, controls = 41). Phase II implements longitudinal monitoring of rhGH recipients (*n* = 61) with quarterly growth parameter assessments. This will allow for the assessment of the potential association between the microbial/SCFA characteristic spectrum and treatment outcomes, such as post-treatment height growth velocity and IGF-1 levels. It is hypothesized that this study will elucidate the connection between gut microbial ecology, SCFA metabolism, and the efficacy of rhGH treatment. Moreover, the findings will lay a preclinical foundation for the development of adjunctive microbiota-targeted strategies. By modulating the gut microbiota and related metabolic pathways, these strategies may enhance the therapeutic effects of growth hormone, potentially leading to improved growth outcomes for children with short stature.

## 2 Materials and methods

### 2.1 Ethical statement

The Ethics Committee of the Children's Hospital, Shanghai Jiao Tong University School of Medicine has given its approval for all recruitment and research procedures (Ethics Approval 2020RY068-E01). Before taking part in the study, parents or legal guardians provided their written informed consent, and minors likewise expressed their agreement.

### 2.2 Study design and inclusion criteria

The study was designed and conducted in 2 phases. A total of 69 children with short stature and 41 age- and sex-matched healthy volunteers were recruited. After a rigorous screening process, 65 children with short stature who met the inclusion criteria were finally selected for the study. Inclusion criteria (23, 24): ① Height below the 3rd percentile (−1.88 SD) or −2 SD of healthy children of the same age and gender; ② Annual growth rate <7 cm/year (less than 3 years old); <5 cm/year (3 years old and 1 year before puberty); <6 cm/year (puberty). ③ Short stature with youthful appearance; ④ Normal cognitive development; ⑤ Bone age less than chronological age (25) (assessed using the TW2 method). Exclusion criteria: Participants will be excluded if they have hypothyroidism (elevated TSH and decreased FT4), Turner syndrome (confirmed by karyotype analysis showing 45, X, or other X chromosome abnormalities), SHOX gene mutations (identified by molecular genetic testing), malnutrition (history of inadequate caloric intake and nutritional deficiencies), or renal insufficiency. Abnormal renal function tests and GFR below the normal range for age, IUGR (birth weight and length below the 3rd percentile for gestational age and history of prenatal growth restriction), or skeletal dysplasia (bone age assessment and arm span-to-height ratio outside the normal range for age and sex). To minimize the impact of diet on gut microbiota, participants were recruited from the eastern region of China near Shanghai, where dietary patterns are similar. Those following a completely vegetarian diet were excluded. The first phase was a case-control study. The objective of this phase was twofold: first, to analyze the gut microbiota characteristics of GHD or ISS compared with normal controls, and second, to investigate the effect of rhGH treatment on the gut microbiota and SCFA production in children with short stature.

**Figure 1:**
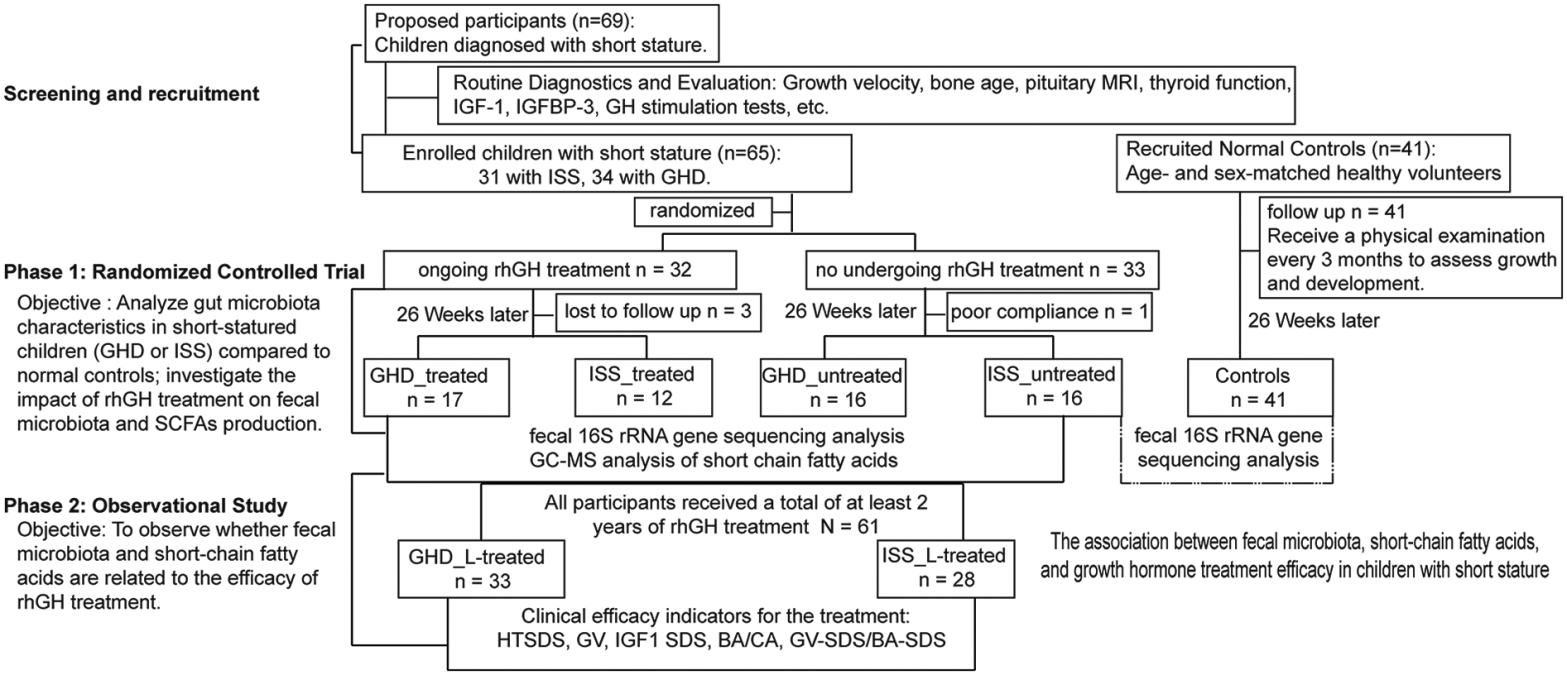
Participant flow and group assignments. Phase 1: 69 children with short stature and 41 healthy volunteers were recruited. After screening, 65 children were randomized into treatment (*n* = 32) and non-treatment (*n* = 33) groups. After 26 weeks, 3 treatment and 1 non-treatment participants dropped out. The treatment group included 17 GHD and 12 ISS patients; the non-treatment group had 16 GHD and 16 ISS patients. The control group comprised 41 healthy volunteers. Phase 2: All short stature patients received rhGH treatment, with 33 GHD and 28 ISS patients completing ≥2 years. Follow-ups every 12 weeks tracked growth and other parameters. GHD, growth hormone deficiency; ISS, idiopathic short stature; IGF-1, insulin growth factor 1; rhGH, recombined human growth hormone; HTSDS, height standard deviation score; GV, height velocity; BA, bone age; CA, chronological age.

In the first phases, the 65 participants with short stature were randomly assigned to either the treatment or non-treatment group. After 26 weeks, 3 cases in the treatment group were lost to follow-up and 1 case in the observation group dropped out due to poor compliance.The treatment group had 17 patients with GHD and 12 with ISS, while the non-treatment group had 16 GHD patients and 16 ISS patients. The normal control group underwent routine medical check-ups every 3 months to assess growth and development. The dosage of growth hormone (Jintropin®) ranged from 0.025 mg to 0.05 mg/kg/day, adjusted by the physician. After 26 weeks, all patients with short stature underwent gut microbiota and SCFA testing, while the normal control group underwent fecal microbiota testing only. The second phase was an observational study. The objective of this phase was to observe whether there was an association between gut microbiota and SCFAs and the efficacy of rhGH treatment. In this phase, all patients with short stature received rhGH treatment, with 33 patients in the GHD group and 28 patients in the ISS group completing at least 2 years of treatment. During the treatment period, patients were followed every 12 weeks to monitor growth velocity, IGF-1 levels, bone age, and other parameters. Growth velocity in the growth velocity percentile of age-matched children was used as an assessment index to explore the association between fecal microbiota, SCFA, and the effect of growth hormone treatment. Height was measured in the morning while standing in front of a calibrated wall stadiometer; during each clinical visit, height was measured 3 times and the average was calculated. Growth velocity: Growth velocity (cm/yr) = (Height2-Height1)/(months between times) × 12. The percentile criteria for height growth velocity used in this study were developed with reference to studies from Beijing, Hong Kong China, Korea, and India, as shown in Supplementary Table S1 (26–29). The flowchart of the study design is shown in Figure 1.

### 2.3 Fecal collection and preparation

Participants were asked not to take any probiotics or antibiotics for 4 weeks prior to fecal sampling. Participants' legal guardians were instructed to collect fresh stool samples using the stool collection device provided in the restroom. Fresh fecal samples were collected on-site on the morning of the scheduled visit and immediately transferred to sterile tubes before being returned to the researcher and frozen at −80°C for DNA extraction and mass spectrometry analysis. In reality, due to the COVID-19 pandemic in Shanghai, some samples were collected at home by parents as needed, shipped to the researchers via dry ice cold chain, and stored at −80°C.

### 2.4 DNA extraction and PCR amplification

Total DNA extraction was performed according to the instructions of the E.Z.N.A.® Soil Kit (Omega Bio-Tek, Norcross, GA, U.S.), and DNA concentration and purity were determined using the NanoDrop 2000, and the quality of DNA extraction was determined using 1% agarose gel electrophoresis; the quality of DNA extractions was determined using 338F (5′-ACTCCTACGGGAGGCAGCAG-3′) and 806R (5′-GGACTACHVGGGGTWTCTAAT-3′) primers for PCR amplification of the V3–V4 variable region, and the 20 µl amplification system contains: 4 µl 5× Fast Pfu buffer, 2 µl 2.5 mM dNTPs, 0.8 µl primer (5 µM), 0.4 µl Fast Pfu polymerase, and 10 ng DNA template.

### 2.5 Library construction and illumina miseq sequencing

A 2% agarose gel recovered PCR products, which were purified using the AxyPrep DNA Gel Extraction Kit (Axygen Biosciences, Union City, CA, USA), rinsed with Tris-HCl, and detected by 2% agarose electrophoresis. Quantification was performed using QuantiFluor™-ST (Promega, USA). The purified fragments were built into libraries using standard procedures on the Illumina MiSeq platform. The steps of library construction were (1) ligation of the “Y” junction, (2) screening to remove junction fragments, (3) enrichment of the library template by PCR, and (4) denaturation to produce single-stranded DNA fragments. High—throughput sequencing of the 16 S rRNA gene was performed using the Illumina MiSeq PE300 platform, which is capable of sequencing thousands of copies simultaneously.

### 2.6 Data quality control and species annotation

Microbiome bioinformatics processing was conducted following the standardized QIIME2 (30) pipeline (v2023.9) as outlined in the Atacama Soil Microbiome tutorial (31). Demultiplexed sequence data were imported using the q2-toolsimport plugin and subjected to rigorous quality control through the q2-DADA2 workflow, implementing denoising (truncation length: forward 290 bp, reverse 210 bp), dereplication, chimera detection (consensus method), and amplicon sequence variant (ASV) table generation. Taxonomic assignment was performed via the q2-feature classifier with a 99% similarity threshold against the Greengenes 13_8 reference database (V3–V4 hypervariable region; region-specific curation based on 338F/806R primer sequences). Potential contamination from host-associated (mitochondria) and dietary (chloroplast) sources was systematically excluded using q2-feature-table filtering. Differential abundance analysis employed parametric (ANOVA) and nonparametric (Kruskal–Wallis) tests for normally/non-normally distributed features, supplemented by linear discriminant analysis effect size (LEfSe) modeling (LDA score > 2.5, α = 0.05) to identify taxon-specific biomarkers across clinical subgroups.

### 2.7 Metabolomics of fecal SCFAs in children with short stature

#### 2.7.1 Standard preparation

Weigh the acetic, propionic, butyric, isobutyric, valeric, isovaleric, and hexanoic acid standards and prepare eight mixed standard concentration gradients of 0.1 μg/ml, 0.5 μg/ml, 1 μg/ml, 5 μg/ml, 10 μg/ml, 20 μg/ml, 50 μg/ml, and 100 μg/ml using ethyl acetate. For GC-MS detection, combine 600 μl of the standard with 25 μl of 4-methylpentanoic acid at a final concentration of 500 μM as an internal standard. Fill the injection bottle with 1 μl and use a 10:1 shunt ratio for injection.

#### 2.7.2 Metabolite extraction

For metabolite extraction, thaw the sample on ice and transfer 30 mg into a 2 ml centrifuge tube. For GC-MS detection, add 900μl of 0.5% phosphoric acid resuspension, shake and mix for 2 min, and centrifuge at 14,000 g for 10 min. Take 800μl of the supernatant, add the same volume of ethyl acetate extraction, shake and mix for 2 min, and centrifuge at 14,000 g for 10 min. Finally, take 600μl of the upper layer of the organic phase and add a final concentration of 500μM 4-methylglutaric acid as an internal standard, mix well, and transfer to the injection vial for GC-MS detection.

#### 2.7.3 Chromatography and mass spectrometry analysis

(1) Gas chromatography parameters: The samples were separated using an Agilent DB-WAX capillary column (30 m × 0.25 mm ID × 0.25 μm) in gas chromatography. The temperature was gradually increased from 90°C to 250°C over a period of 2 min. The carrier gas was helium at a flow rate of 1.0 ml per minute. The sample cohort was configured with a QC sample at each interval of a certain number of experimental samples, which was used to detect and evaluate system stability and repeatability. (2) Mass Spectrometry Analysis: The Agilent 7890A/5975C gas mass spectrometer was used for mass spectrometry analysis. The injection port was 250℃, the ion source was 230℃, the transmission line was also 250℃, and the quadrupole was 150℃. Electron bombardment ionization (EI) source, full sweep and SIM scanning modes, electron energy of 70 eV. (3) Data processing: The retention time and chromatographic peak area were extracted using MSD ChemStation software. The standard curve was plotted, and the amount of short-chain fatty acids in the samples was calculated.

### 2.8 Data analysis and statistics

Microbial community analyses were performed using QIIME2 (v2023.9) with rarefaction to 95% of the minimum sequencing depth (20,890 reads per sample). Alpha diversity was evaluated using the Shannon index and Peillou evenness, with between-group differences assessed via Kruskal–Wallis tests followed by Dunn's *post-hoc* comparisons with two-stage step-up method of Benjamini, Krieger, and Yekutiel correction. Beta diversity was calculated based on Bray-Curtis dissimilarity, visualized through principal coordinates analysis (PCoA) using the Wekemo Bioincloud platform (v3.2) (32), and statistical significance of group differences was determined by PERMANOVA with 999 permutations. Differential abundance analysis at the genus level incorporated both LEfSe (LDA score > 2.5, Benjamini-Hochberg adjusted *P* < 0.05) and the nonparametric ANCOM-BC model. Functional potential prediction was conducted using PICRUSt2 (v2.5.0) with KEGG Orthology pathway enrichment analysis (FDR <0.1 threshold). Clinical-microbiome integration analysis was performed through redundancy analysis (RDA) in Canoco5 using 500 Monte Carlo permutations. Statistical analyses were primarily executed in R (v4.3.1) and GraphPad Prism 9.3.1. Continuous variables are presented as median (interquartile range, IQR), with parametric *t*-tests or nonparametric Mann–Whitney *U*-tests selected based on normality assessment via Shapiro–Wilk test (*P* > 0.05 indicating normal distribution).

## 3 Results

### 3.1 Baseline characteristics of study participants

The subjects included in this study comprise the baseline characteristics of patients with GHD, ISS, and those in the control group, as shown in Table 1. The data indicate that the sample sizes for the 3 groups were 33, 28, and 41, respectively. The mean ages (CA, chronological age) for each group were 7.90 ± 2.89 years, 9.09 ± 2.89 years, and 8.21 ± 1.43 years (*P* = 0.15). The gender distribution was balanced, with males comprising 51.5%, 46.4%, and 48.7% of the groups, respectively (*P* = 0.92). The mean weight exhibited significant disparities among the GHD group (23.25 ± 7.39 kg), the ISS group (25.95 ± 11.26 kg), and the control group (30.37 ± 7.15 kg) (*P* = 0.002). Both the GHD and ISS groups demonstrated significantly lower mean weights compared to the control group (**P < 0.01). In terms of height standard deviation (SD), the GHD group (−2.13 ± 0.36) and the ISS group (−2.14 ± 0.37) were significantly lower than the control group (0.54 ± 0.57, *P* = 0.00), while the differences in BMI were not statistically significant (*P* = 0.13). A further analysis of mid-parental height (MPH) reveals that the male GHD group (170.38 ± 3.92 cm) and the ISS group (167.87 ± 5.04 cm) are significantly lower than the control group (172.57 ± 4.43 cm, *P* = 0.00). A similar trend was observed in the female GHD group (157.17 ± 5.64 cm) and the ISS group (157.44 ± 3.63 cm), which were both significantly lower than the control group (163.16 ± 4.06 cm, *P* = 0.00). The findings suggest that the GHD and ISS groups exhibited substantial disparities in growth parameters (weight, height SD, and MPH) in comparison to the control group. However, the analysis revealed no intergroup heterogeneity in age, gender, and BMI (one-way ANOVA and Tukey's multiple comparison test).

**Table 1:** Baseline characteristics of the study participants.

Demographic characteristics	GHD	ISS	Control	*P* value
n	33	28	41	
CA (year)	7.90 ± 2.89	9.09± 2.89	8.21± 1.43	0.15
Gender, *n* (%)				
Male	17 (51.5)	13 (46.4)	20 (48.7)	0.92 (Chi-square test)
Female	16 (48.4)	15 (53.5)	21 (51.2)	
Weight (kg)	23.25 ± 7.39**	25.95 ± 11.26	30.37 ± 7.15	0.002
Height (SD)	−2.13± 0.36**	−2.14± 0.37**	0.54± 0.57	0.00
BMI	16.33 ±2.64	15.79± 1.92	16.89± 1.99	0.13
MPH (cm)				
Male	170.38± 3.92^△^	167.87 ± 5.04**	172.57± 4.43	0.00
Female	157.17 ± 5.64**	157.44 ± 3.63**	163.16 ± 4.06	0.00

Data are expressed as means ± SD. Statistical differences were analyzed using one-way ANOVA with Tukey's multiple comparison test. Significance levels: *P < 0.05 and **P < 0.01 compared to the control group; ^△^*P* < 0.05 and ^△△^*P* < 0.01 compared to the ISS group. CA, chronological age. MPH, mid-parental height (cm), is calculated as (Mother's height + 13 + Father's height) ÷ 2 for boys and (Father's height—13 + Mother's height) ÷ 2 for girls.

### 3.2 Distinct gut microbiota/SCFA signatures across etiological subtypes of children with short stature

#### 3.2.1 Fecal microbial diversity and richness

**Figure 2:**
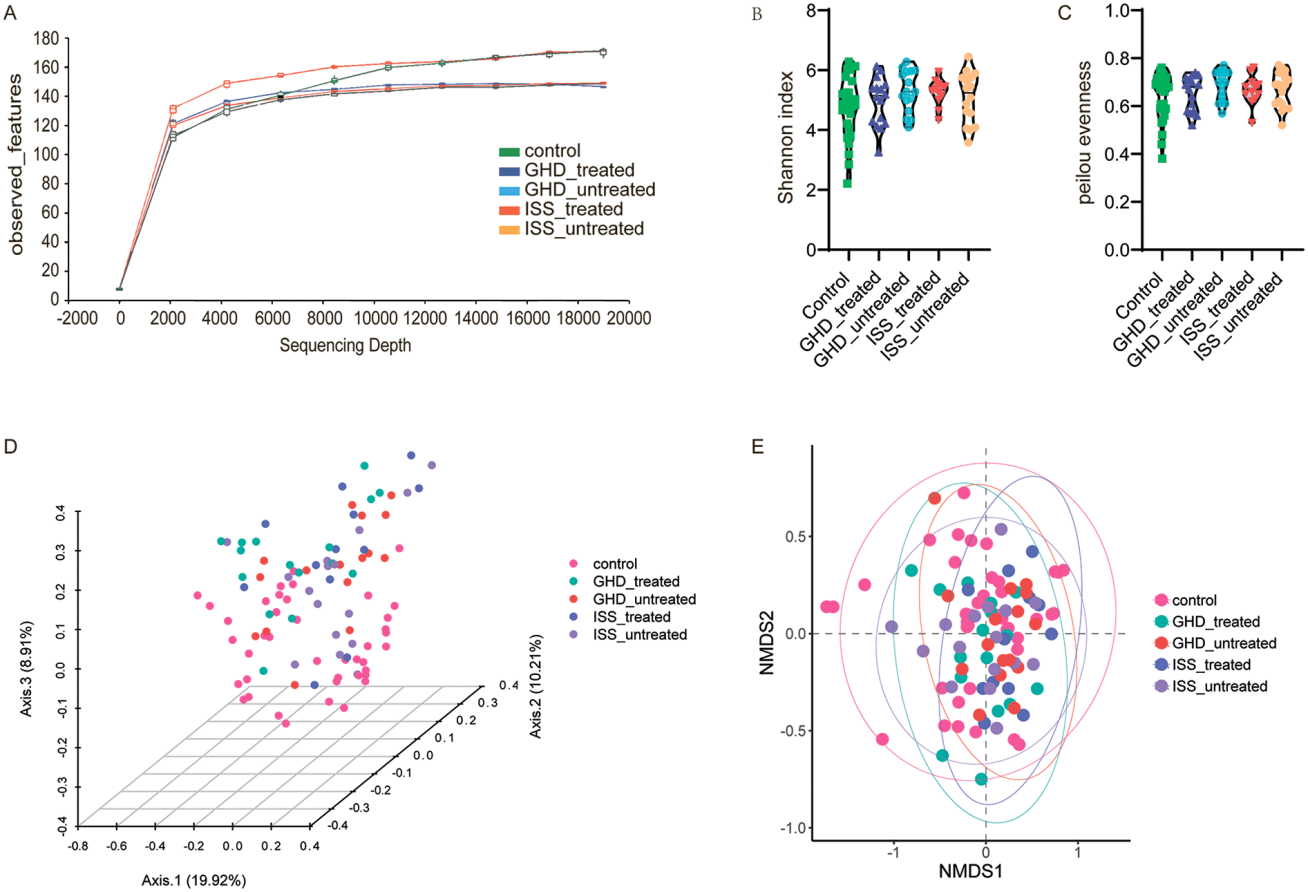
Microbiome diversity analysis. **(A)** Rarefaction curves showing observed features as a function of sequencing depth for control, GHD_treated, GHD_untreated, ISS_treated, and ISS_untreated groups. **(B)** Alpha diversity measured by the Shannon index, with significant differences identified by Kruskal–Wallis test and Benjamini, Krieger, and Yekutieli correction. **(C)** Pielou evenness index, analyzed similarly. **(D)** Principal Coordinate Analysis (PCoA) based on Bray-Curtis dissimilarity, illustrating community structure variation among groups (PERMANOVA: *R*^2^ = 0.067, *F* = 3.524, *P* = 0.001). **(E)** Non-metric Multidimensional Scaling (NMDS) plot showing community composition differences, with similar PERMANOVA results (*F* = 3.524, *P* = 0.001).

Fecal microbiota profiling was conducted on 61 children with short stature and 41 normal-height controls using Illumina MiSeq sequencing (Illumina Inc., USA). A total of 5,629,967 raw sequences were generated, with per-sample counts ranging from 34,253 to 131,510 (median: 45,710). Sequences were processed through the DADA2 pipeline for primer removal, quality filtering, and chimera elimination, retaining a median of 28,468 high-quality sequences per sample. The processed sequences exhibited a median length of 413.92 bp (range: 264–431 bp), yielding 6,422 amplicon sequence variants (ASVs). Rarefaction curves confirmed sufficient sequencing depth across all samples (Figure 2A). Alpha diversity analysis revealed no significant between-group differences in Pielou evenness (Kruskal–Wallis test, *P* = 0.506) or Shannon index (Kruskal–Wallis test, *P* = 0.436,) (Figures 2B,C). In contrast, beta diversity analysis demonstrated distinct microbial community structures. Principal coordinates analysis (PCoA) based on Bray-Curtis dissimilarity showed significant separation between groups (PERMANOVA: *R*^2^ = 0.067, *F* = 3.524, *P* = 0.001), with the first two axes explaining 19.92% and 10.21% of total variance, respectively (Figures 2D,E). Notably, while significant structural differences were observed, the short stature group showed no alterations in overall gut microbial abundance or diversity.

#### 3.2.2 Characteristics of fecal microbiota in children with short stature

**Figure 3:**
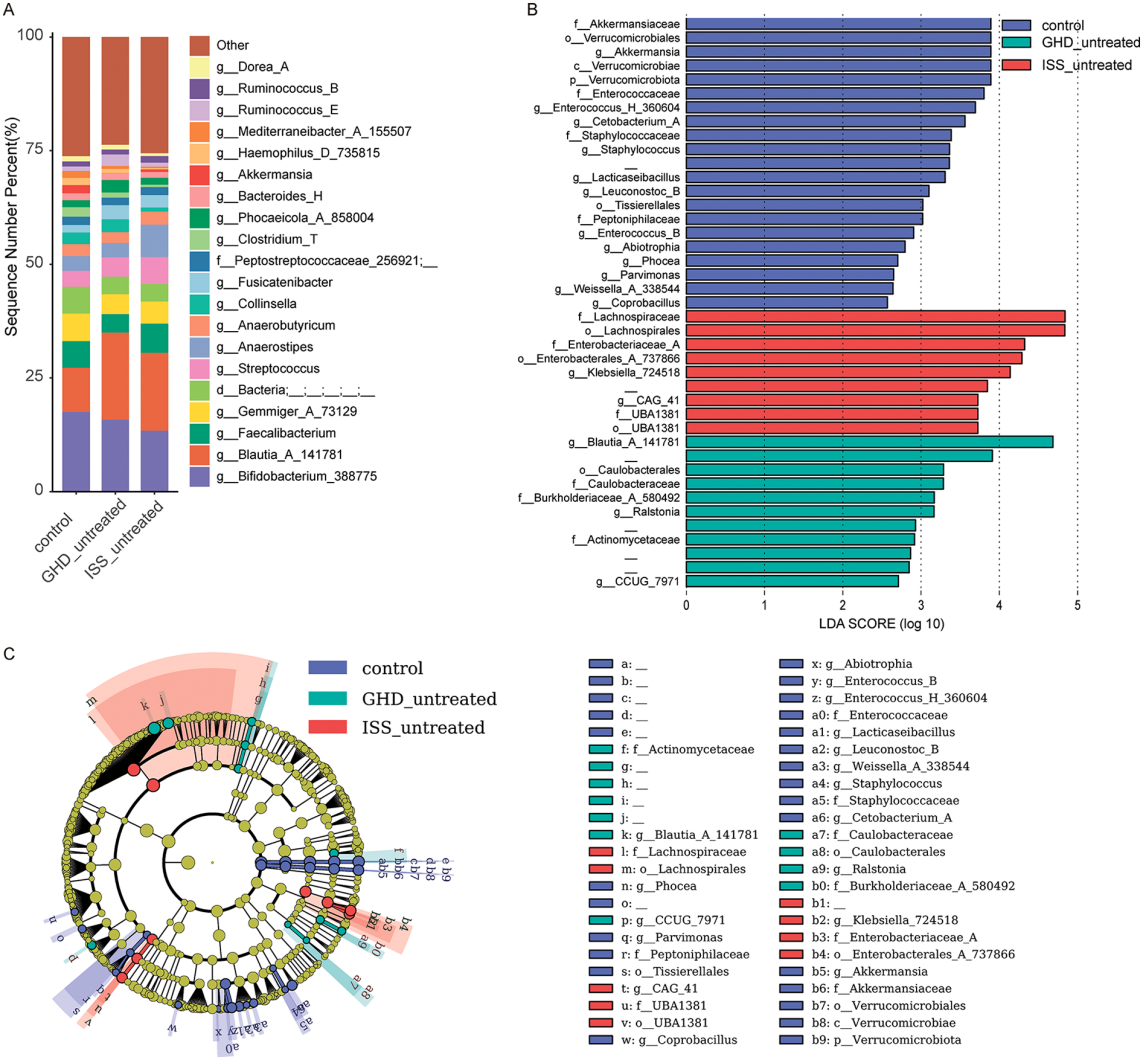
Microbiome composition and differential abundance analysis. **(A)** Bar chart showing the relative abundance of bacterial genera in control (*n* = 41), GHD_untreated (*n* = 16), and ISS_untreated (*n* = 16) groups. **(B)** LEfSe LDA score plot highlighting differentially abundant taxa between the groups, with LDA scores > 2.5 indicating significant differences. **(C)** LEfSe cladogram visualizing taxonomic distribution and abundance differences among the groups, where nodes represent taxa and colors indicate group-specific enrichment. Only taxa with LDA scores > 2.5 are displayed.

To investigate associations between gut microbiota and childhood short stature, and to characterize microbial differences among untreated growth hormone deficiency (GHD_untreated, *n* = 16), idiopathic short stature (ISS_untreated, *n* = 16), and healthy controls (*n* = 41), we analyzed fecal microbiota composition. Untreated growth disorders exhibited reduced abundances of beneficial butyrate-producing genera (e.g., *Faecalibacterium*) and elevated levels of potential pathobionts, consistent with disease phenotypes. Healthy controls demonstrated higher relative abundances of health-associated taxa, including *Bifidobacterium_388775* (Actinobacteriota: 17.5% vs. 15.8% in GHD and 13.4% in ISS) and *Akkermansia* (Verrucomicrobiota: 1.8% vs. near-absent levels in GHD 0.03%). In contrast, untreated groups displayed enrichment of dysbiosis-linked taxa: ISS showed higher *Streptococcus* (Firmicutes_D: 5.8% vs. 3.5% in controls) and reduced *Clostridium_T* (Firmicutes_A: 0.5% vs.2.1%), while GHD was enriched with *Blautia_A_141781* (Firmicutes_A: 19.2% vs. 9.7% in controls), and ISS harbored more *Anaerostipes* (Firmicutes_A: 7.1% vs. 3.3% in controls). Notably, the pro-inflammatory genus *Collinsella* (Actinobacteriota) was elevated in GHD (2.8%) compared to ISS (0.9%). LEfSe analysis (Figure 3B) identified control-enriched taxa such as Akkermansiaceae (LDA = 3.8) and Verrucomicrobiales (LDA = 3.8), while GHD/ISS groups were enriched with Actinomycetaceae (LDA = 2.8) and Enterobacteriaceae (LDA = 4.3). Phylogenetic analysis (Figure 3C) further revealed control-associated clusters within Firmicutes (e.g., Enterococcaceae) and Verrucomicrobia (e.g., *Akkermansia*), contrasting with GHD/ISS-linked Proteobacteria (e.g., *Klebsiella_724518*) and Actinobacteria (e.g., Actinomycetaceae). These findings suggest that untreated growth disorders are associated with depletion of beneficial taxa (e.g., *Akkermansia*, *Faecalibacterium*) and expansion of pro-inflammatory or opportunistic genera, implicating microbiota dysregulation in disease pathophysiology. All species exhibiting relatively high abundance or distinctive characteristics, along with their full taxonomic classifications (including kingdom, phylum, class, order, family, and genus) for those confidently identified to the genus level, are provided in Supplementary Table S2.

#### 3.2.3 Predicted metabolic pathways in the fecal microbiota of children with short stature

**Figure 4:**
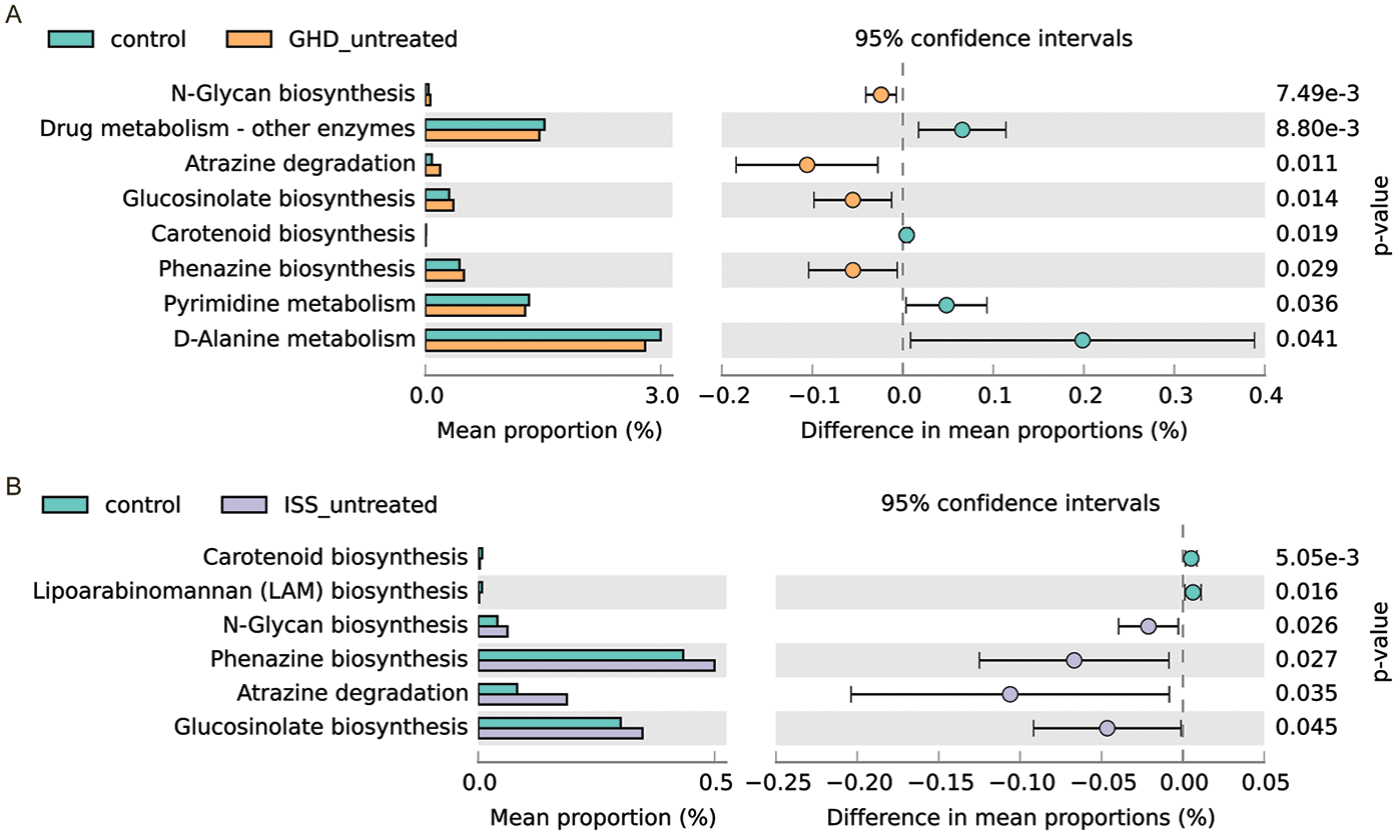
Comparison of predicted Gut microbiome metabolic pathways. **(A)** Comparison between control (*n* = 41) and GHD_untreated (*n* = 16) groups. **(B)** Comparison between control (*n* = 41) and ISS_untreated (*n* = 16) groups. For both panels, the left side shows the mean proportion of each metabolic pathway, and the right side displays the difference in mean proportions along with 95% confidence intervals. Statistical significance was assessed using Welch's *t*-test (CI method: Welch's inverse, 0.95).

Comparative analysis of predicted microbial metabolic pathways revealed distinct dysregulations in untreated ISS and GHD groups compared to controls (Figure 4). Both ISS and GHD groups demonstrated significant enrichment in Glucosinolate biosynthesis (ISS: +0.046%, *P* = 0.045; GHD: +0.055%, *P* = 0.014), Phenazine biosynthesis (ISS: +0.067%, *P* = 0.027; GHD: +0.056%, *P* = 0.029), Atrazine degradation (ISS: +0.106%, *P* = 0.035; GHD: +0.106%, *P* = 0.011), and N-Glycan biosynthesis (ISS: +0.021%, *P* = 0.026; GHD: +0.024%, *P* = 0.007), alongside reduced Carotenoid biosynthesis (ISS: −0.005%, *P* = 0.005; GHD: −0.004%, *P* = 0.019). Unique to ISS was diminished Lipoarabinomannan biosynthesis (−0.006%, *P* = 0.016), while GHD specifically showed suppressed D-Alanine metabolism (−0.199%, *P* = 0.041) and Drug metabolism (−0.065%, *P* = 0.009). These metabolic shifts suggest microbial pathway imbalances in both conditions, characterized by enhanced xenobiotic detoxification and glycan processing, concurrent with deficiencies in nutrient-associated pathways (e.g., carotenoids, D-alanine), potentially disrupting host metabolic homeostasis, immune regulation, and growth-related biological processes.

#### 3.2.4 Characteristics of SCFA metabolism in children with short stature

Based on previous findings of decreased beneficial SCFA-producing genera (e.g., *Faecalibacterium*, *Bacteroides_H*, *g_Collinsella,* and *g_Bacteroides_H*) and dysbiosis in untreated short stature, we investigated whether fecal SCFA levels, particularly propionate, butyrate, and acetate, which are important for gut and metabolic health, correlate with growth impairment. Comparative analysis using Mann–Whitney *U*-tests or *t*-tests (assuming normality) found no significant changes (*P* > 0.05) in acetic, butyric, hexanoic, isobutyric, isovaleric, propionic, or valeric acid levels between untreated GHD and ISS groups (Supplementary Tables S3). Despite significant dysbiosis in the untreated short stature groups, fecal SCFA levels did not differ significantly between the GHD and ISS cohorts, implying that growth hormone insufficiency is not directly related to SCFA production. Further investigation is needed to discover whether exogenous rhGH treatment alters host-microbe interactions or gut microbial metabolic pathways.

### 3.3 Effects of rhGH therapy on fecal microbiome and SCFA metabolism in children with short stature

#### 3.3.1 Effects of rhGH therapy on gut microbiota characteristics in children with short stature

**Figure 5:**
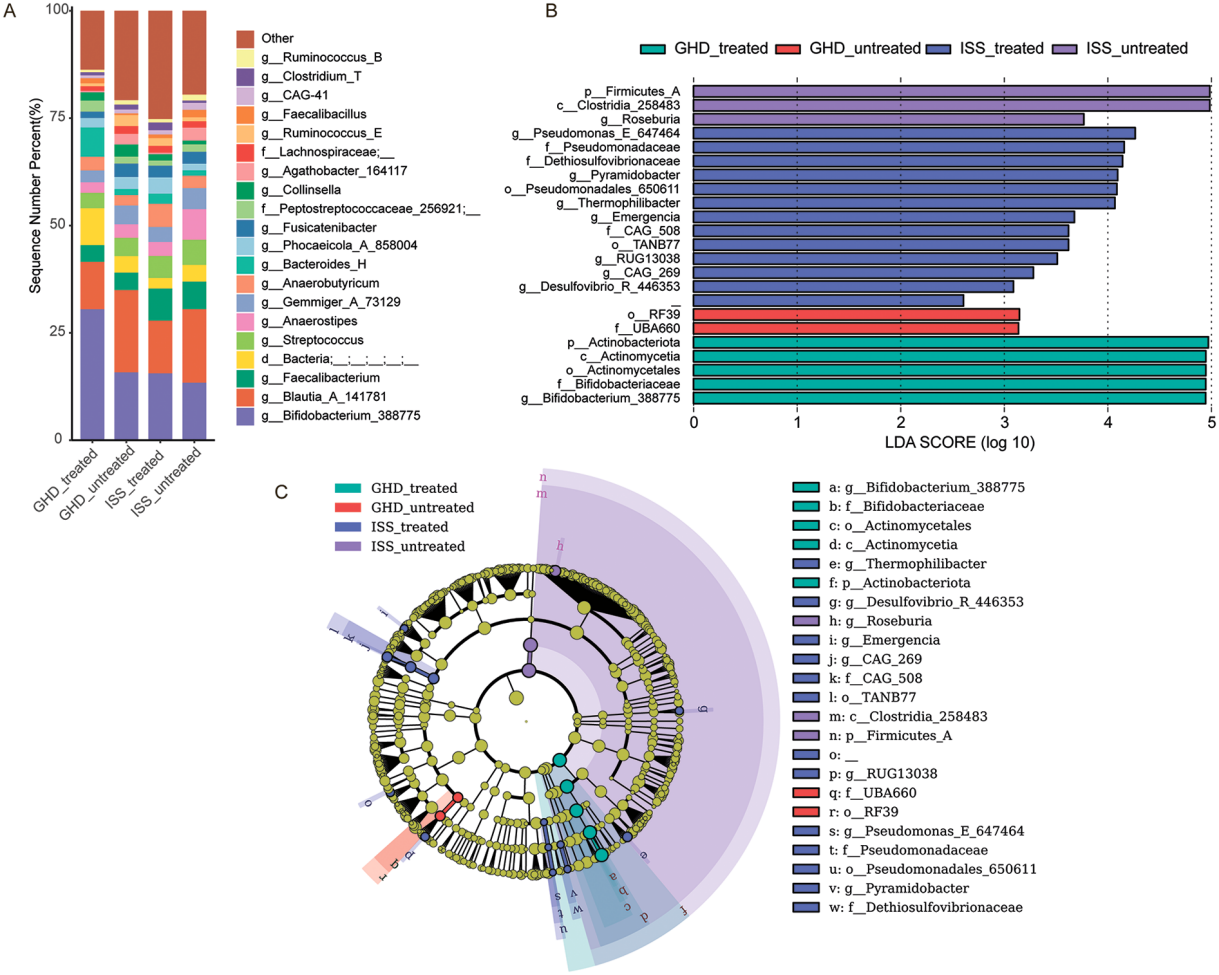
Microbiome composition and differential abundance analysis. **(A)** Bar chart showing the relative abundance of bacterial genera in GHD-treated (*n* = 17), GHD-untreated (*n* = 16), ISS-treated (*n* = 12), and ISS-untreated (*n* = 16) samples. Each bar represents the percentage of sequences assigned to different genera. **(B)** LEfSe (Linear Discriminant Analysis Effect Size) LDA score plot highlighting differentially abundant taxa between the groups. Taxa with an LDA score greater than 2.5 are considered statistically significant, and the length of the bar indicates the magnitude of the difference. **(C)** LEfSe cladogram visualizing the taxonomic distribution of differentially abundant taxa with an LDA score greater than 2.5. Nodes represent taxa, with the size of the node indicating abundance and the color representing the group with higher abundance. Significant taxa are highlighted with colored circles and lines.

Previous studies have shown significant structural changes in the gut microbiota of patients with GHD/ISS, such as a decrease in beneficial bacteria (e.g., *Akkermansia*) and an increase in opportunistic pathogens (e.g., Enterobacteriaceae), suggesting a potential link between the gut microbiota and growth hormone. This study further compared and evaluated the gut microbiota composition of treated and untreated GHD/ISS patients to investigate these changes. Taxonomic composition (Figure 5A) and LDA scores (Figure 5B) revealed distinct microbial signatures between groups. The treated GHD cohort showed increased abundance of *Bifidobacterium_388775* (Actinobacteriota: 30.5% vs. 15.8% in untreated GHD and 13.4% in untreated ISS), a probiotic genus associated with gut health. In contrast, untreated groups exhibited enrichment of *Blautia_A_141781* (Firmicutes_A: 19.2% in untreated GHD vs. 11.0% treated) and *Streptococcus* (Firmicutes_D: 5.8% in untreated ISS vs. 5.1% treated), taxa linked to dysbiosis. The ISS-treated group demonstrated elevated *Faecalibacterium* (Firmicutes_A: 7.5% vs. 6.4% untreated), a butyrate producer, while untreated cohorts harbored higher *Collinsella* (Actinobacteriota: 2.8% vs. 0.9%), a genus implicated in metabolic dysfunction.

#### 3.3.2 Effects of rhGH treatment on predicted metabolic pathways in the fecal microbiota of children with short stature

**Figure 6:**
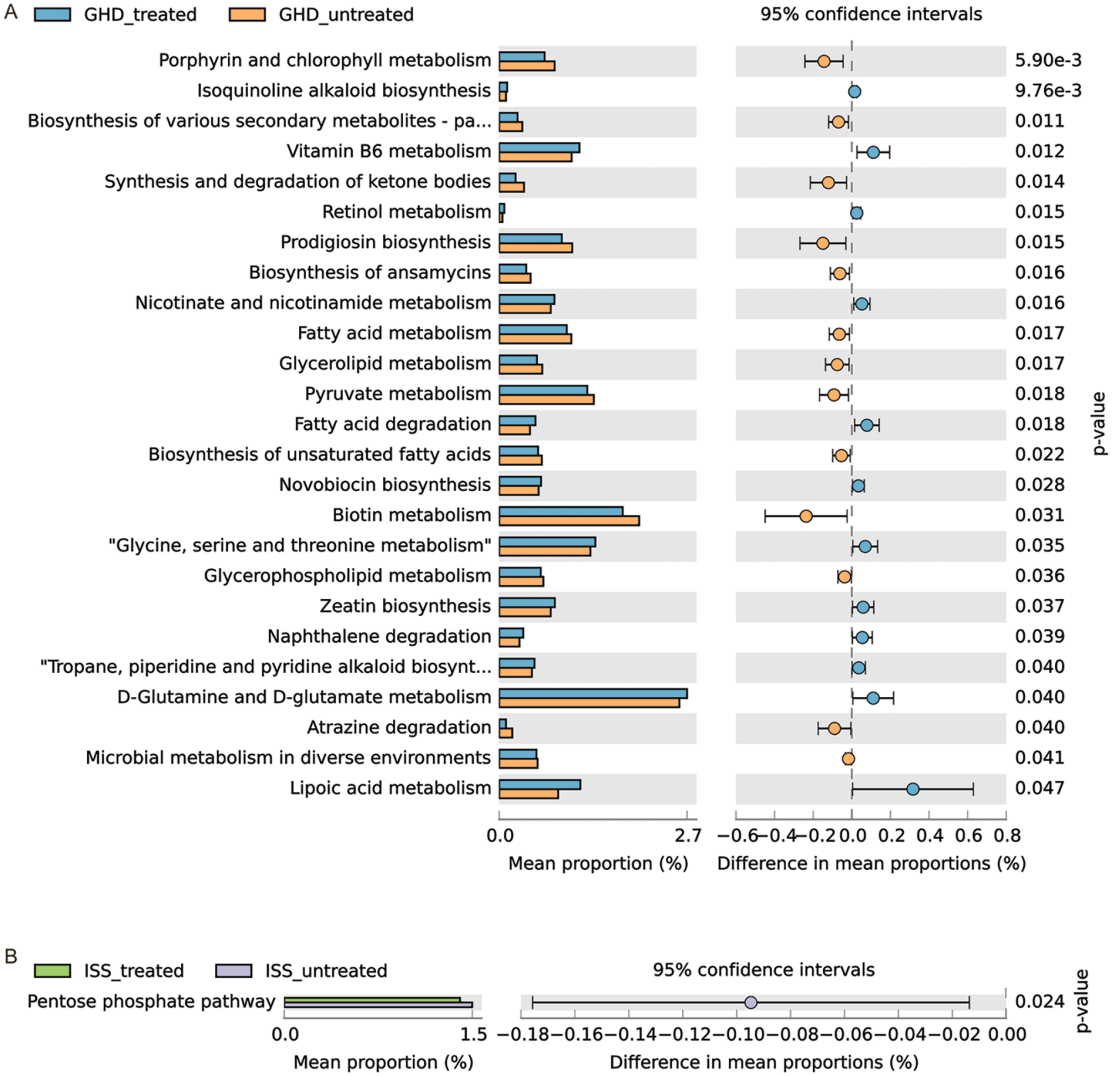
Comparison of predicted gut microbiome metabolic pathways. **(A)** Comparison between GHD-treated (*n* = 17) and GHD-untreated (*n* = 16) groups. **(B)** Comparison between ISS-treated (*n* = 12) and ISS-untreated (*n* = 16) groups. The mean proportions of each metabolic pathway are represented by bars, with the differences in mean proportions between groups shown alongside 95% confidence intervals. Statistical significance was assessed using Welch's *t*-test, and confidence intervals were calculated using Welch's inversion method. *P*-values for each pathway are indicated.

Comparative analysis of predicted metabolic pathways demonstrated distinct alterations between GHD-treated and untreated cohorts (Figure 6A). The “glycine, serine, and threonine metabolism” pathway showed significantly higher relative abundance in treated subjects (1.37% vs. 1.31%, *P* = 0.035), while “fatty acid degradation” exhibited a similar increasing trend (0.52% vs. 0.44%, *P* = 0.018). Conversely, treatment-associated suppression was observed in lipid metabolism pathways, particularly in “synthesis and degradation of ketone bodies” (0.24% vs. 0.36%, *P* = 0.014) and “prodigiosin biosynthesis” (0.90% vs. 1.05%, *P* = 0.015). Notably, “biotin metabolism” displayed marked downregulation in the treatment group (1.77% vs. 2.00%, *P* = 0.031), whereas “nicotinate and nicotinamide metabolism” demonstrated enhanced activity post-intervention (0.79% vs. 0.74%, *P* = 0.016). In the ISS-treated group, pentose phosphate metabolic pathways demonstrated a modest decrease compared to the ISS-untreated group (1.37% vs. 1.46%, *P* = 0.023). The present findings suggest that the administration of rhGH to the ISS significantly impacts carbohydrate metabolism and the pentose phosphate pathway. Collectively, these findings indicate that rhGH treatment modulates amino acid, lipid, and cofactor metabolism while altering secondary metabolism biosynthesis, potentially reflecting therapeutic effects on metabolic homeostasis.

#### 3.3.3 The effects of rhGH therapy on fecal SCFA metabolism

**Figure 7:**
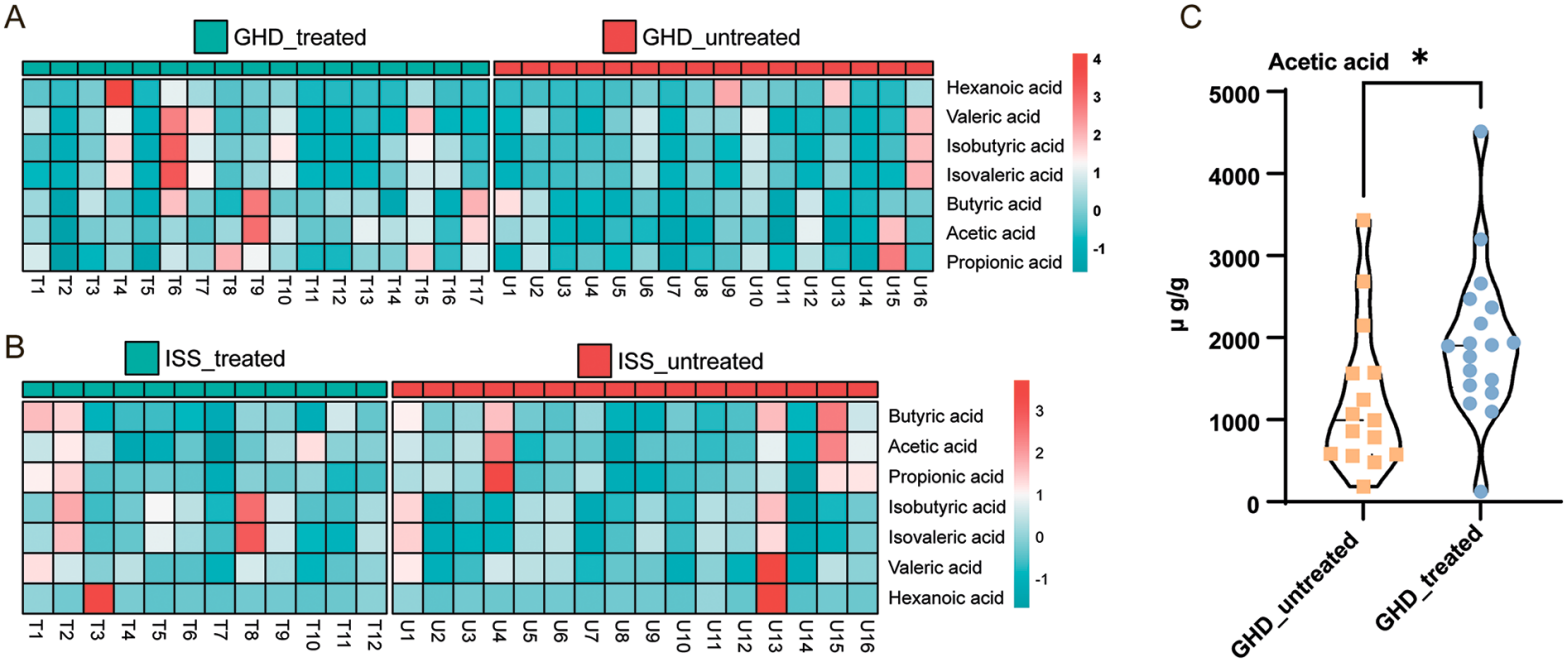
Comparison of short-chain fatty acids (SCFAs) in GHD and ISS subjects. **(A)** GHD Treated vs. GHD Untreated: Heatmap showing the relative abundance of six SCFAs (acetic, propionic, butyric, isobutyric, isovaleric, and valeric acids) in GHD-treated (*n* = 17) and GHD-untreated (*n* = 16) groups. The color gradient from light blue to red indicates low to high abundance. **(B)** ISS Treated vs. ISS Untreated: Heatmap comparing the SCFA profiles in ISS-treated (*n* = 12) and ISS-untreated (*n* = 16) groups, using the same color gradient. **(C)** Acetic Acid Concentration Comparison: Violin plot showing the distribution of acetic acid concentrations between GHD-untreated and GHD-treated groups, with a significant difference indicated by an unpaired *t*-test (*P* = 0.037), denoted by an asterisk (*).

Based on the metagenomic prediction of metabolic pathways implicating lipoic acid metabolism as a gut microbiota-associated pathway closely linked to short stature and growth hormone therapy, we further investigated the correlations between fecal SCFAs and short stature and rhGH treatment. To assess the impact of rhGH therapy on fecal SCFA profiles in children with GHD and ISS, we conducted comparative analyses of SCFA levels between rhGH-treated and untreated cohorts. Chromatographic separation of SCFA standards demonstrated optimal resolution with sharp, symmetrical peaks in the total ion current (TIC) chromatogram, enabling reliable mass spectrometric quantification (Supplementary Figure S1). The heatmap data (Figure 7A) suggests distinct trends in SCFA profiles between GH-treated (T) and untreated (U) GHD groups: GH-treated samples show elevated propionic acid (e.g., T22: 2.049 vs. U15: 2.723) and butyric acid (T17: 2.064 vs. U15: −0.098) in specific cases, while untreated groups exhibit higher branched-chain acids (e.g., U16: isobutyric 1.918 vs. T4: 1.606). Hexanoic acid displays outliers (T4: 4.143, U9: 2.154), suggesting potential treatment-related modulation. However, variability is significant, with treated and untreated groups overlapping in acetic acid (e.g., T9: 2.969 vs. U15: 1.851) and valeric acid (T6: 2.688 vs. U10: 1.145). Overall, rhGH treatment may selectively enhance certain SCFAs (e.g., propionate, butyrate) but does not uniformly suppress branched-chain acids, highlighting context-dependent effects. GH-treated ISS samples (T) show mixed effects on SCFA profiles compared to untreated (U): propionic acid is elevated in some treated samples (e.g., T2: 1.347 vs. U4: 3.400), but untreated groups also display outliers (U13: hexanoic acid 3.588). Butyric acid varies widely (T1: 1.618 vs. U15: 2.421), with no consistent treatment trend. Branched-chain acids (isobutyric/isovaleric) are suppressed in some treated samples (T1: −0.118 vs. U1: 1.381) but elevated in others (T8: 2.639). Hexanoic acid exhibits extreme variability (T3: 3.423 vs. U13: 3.588). Quantitative analysis (Figure 7C) revealed that acetic acid showed statistically significant elevation following rhGH treatment in GHD patients (*t* = 2.176, *df* = 31, *P* = 0.037). Overall, rhGH treatment does not uniformly modulate SCFAs in patients with short stature, with context-specific increases or decreases and notable outliers complicating interpretation. To accurately assess the impact of rhGH treatment on short-chain fatty acid metabolism in the gut, it is necessary to further increase the sample size and eliminate the influence of confounding factors.

### 3.4 The association between gut microbiota and SCFA metabolism in children with short stature and the clinical efficacy of rhGH treatment

The previous results show that children with ISS and GHD have distinct gut microbiota profiles and metabolic alterations. The rhGH therapy can partially restore the microbiota by increasing beneficial bacteria and reducing pathobionts, but its effects on SCFA profiles are inconsistent. Further research is needed to explore the links between gut microbiota, SCFA metabolism, and rhGH treatment efficacy. To investigate the association of gut microbiota and SCFAs with rhGH therapeutic outcomes, we established a primary scoring system using first-year growth velocity in rhGH-treated children relative to age- and sex-matched reference standards (24, 26, 33) (see Supplementary Table S1). Following this comparative analysis, post-treatment growth velocities were stratified into three percentile-based categories: high responders (>97th percentile), moderate responders (90th–97th percentile), and standard responders (75th–90th percentile). This stratification framework facilitated subsequent studies of microbial and metabolic parameters across the different response groups.

#### 3.4.1 Fecal microbiota characteristics of children receiving rhGH with different percentile growth velocities

**Figure 8:**
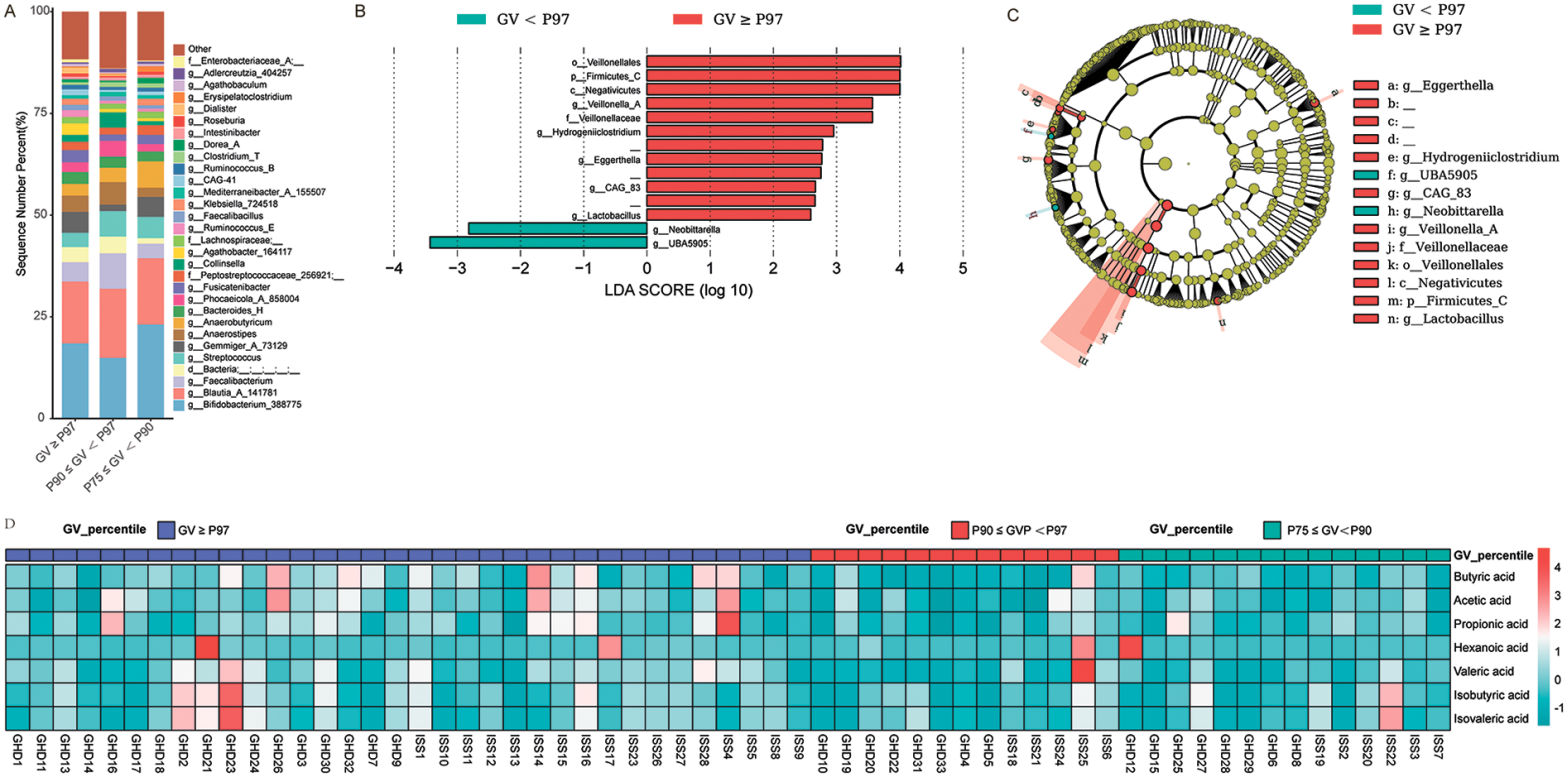
Comparison of microbial composition and metabolic profiles across different growth velocity (GV) percentiles. **(A)** Bar chart showing the percentage distribution of bacterial taxa across three GV percentile groups: GV ≥ P97 (red), P90 ≤ GV < P97 (blue), and P75 ≤ GV < P90 (cyan). Each bar represents the relative abundance of various bacterial taxa within each GV percentile group. **(B)** LEfSe LDA score plot highlighting significant differences in bacterial taxa between GV < P97 and GV ≥ P97 groups. The LDA scores (log10) are plotted on the *x*-axis, with higher scores indicating greater differences in abundance. Significant taxa are highlighted in red for GV ≥ P97 and cyan for GV < P97. **(C)** LEfSe cladogram illustrating taxonomic distribution and significant differences in microbial abundance between GV < P97 and GV ≥ P97 groups. The outer ring represents the taxonomic hierarchy, while the inner rings show the abundance of taxa in each group. Significant taxa are highlighted in red for GV ≥ P97 and cyan for GV < P97, with the size of the segments indicating relative abundance. **(D)** Heatmap depicting metabolic profiles associated with different GV percentiles. The heatmap compares the concentration of various metabolites (e.g., butyric acid, acetic acid, propionic acid, hexanoic acid, valeric acid, isobutyric acid) across three GV percentile groups: GV ≥ P97 (red), P90 ≤ GV < P97 (blue), and P75 ≤ GV < P90 (cyan). Color intensity indicates metabolite concentration, with darker shades representing higher concentrations.

Figure 8 systematically characterizes gut microbiota composition and differential abundance patterns associated with growth velocity (GV) percentiles (GV < P97 vs. GV ≥ P97). Figure 8A presents genus-level taxonomic profiles through a stacked bar plot, revealing distinct distribution patterns between groups. Notably, *Bifidobacterium_388775* (Actinobacteriota), *Blautia_A_141781* (Firmicutes_A), and *Streptococcus* (Firmicutes_D) demonstrate elevated relative abundance in the GV ≥ P97 cohort. Conversely, *Faecalibacterium* (Firmicutes_A) and *Gemmiger_A_73129* (Firmicutes_A) predominate in lower GV percentiles (P75–90 and P90–97). The substantial “Other” category across all groups underscores considerable microbial diversity beyond major taxonomic assignments.

**Figure 9:**
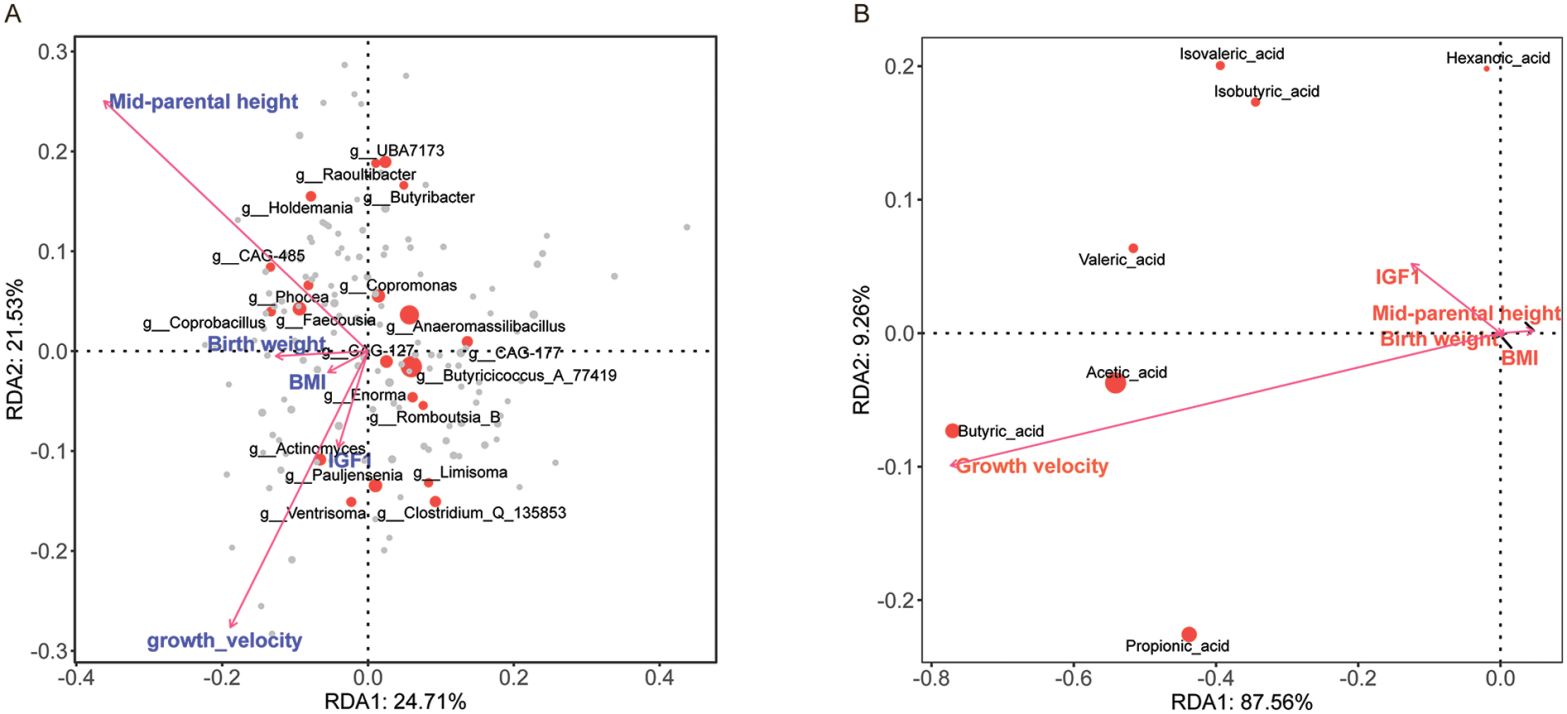
Redundancy analysis (RDA) of clinical efficacy indicators and fecal microbiota/SCFAs. **(A)** RDA1 (24.71% variance) and RDA2 (21.53% variance) showing associations between clinical factors (e.g., growth velocity, birth weight, mid-parental height, BMI, IGF1) and microbial genera. Vectors indicate explanatory variables; positions of genera show relationships with these factors. **(B)** RDA1 (87.56% variance) and RDA2 (9.26% variance) highlighting connections between clinical factors and SCFAs. Vectors represent explanatory variables; positions of SCFAs indicate relationships with these factors. Positive correlations are shown by vectors pointing in the same direction as the genera or SCFAs, while negative correlations are indicated by vectors pointing in opposite directions.

The LEfSe analysis (Figure 8B) identifies significantly differentiated taxa (LDA > 2.5, *P* < 0.05), with *Veillonella_A* (LDA: 3.78, *P* = 0.014), Negativicutes (LDA: 4.42, *P* = 0.049), and *Eggerthella* (LDA: 3.34, *P* = 0.036) being enriched in GV ≥ P97 samples. The cladogram (Figure 8C) hierarchically illustrates taxonomic relationships, highlighting Firmicutes dominance through distinct clades: *Clostridia_258483* (Lachnospirales/Oscillospirales) and Bacteroidota representatives (*Bacteroides_H, Phocaeicola_A_858004*). Firmicutes_C (Negativicutes) and Firmicutes_D (Bacilli) exhibit differential branching patterns corresponding to GV groupings, with *Lactobacillus* (Firmicutes_D) and *Bifidobacterium* (Actinobacteriota) showing preferential association with higher GV percentiles.

Concurrent analysis (Figure 8D) of fecal SCFAs in rhGH-treated GHD and ISS children indicates growth rate-dependent patterns: Acetic acid: Elevated in the P97 group (GHD1: 0.197; ISS1: 0.722) vs. P75_90 (GHD12: −0.169); Butyric acid: Higher levels in P97 (GHD23: 1.584; ISS16: 1.684) vs. P75_90 (GHD15: −0.707); Hexanoic acid: Significantly increased at P97 (ISS25: 2.947; GHD21: 4.680) compared to lower percentiles; Valeric/isovaleric acids: Decreased in P75_90 (GHD15: −0.997/-0.974) vs. P97 (GHD30: 1.391/0.895). Propionic acid showed minimal variation between groups, although peaks occurred in P97 samples (GHD16: 2.328; ISS4: 3.861). These findings suggest a positive correlation between SCFA abundance (particularly acetic, butyric, and hexanoic acids) and growth velocity, potentially indicating microbiota-mediated metabolic adaptations during rhGH therapy.

#### 3.4.2 Association of rhGH therapy clinical markers with gut microbiota (genus level) and SCFA levels

A redundancy analysis (RDA) (Figure 9A) explored links between microbial community types and clinical efficacy indicators of short stature, using variables like treatment start age, IGF-1, insulin, birth weight, BMI, and post-treatment growth velocity. The analysis showed significant clinical efficacy indicators-microbial associations. Mid-parental height had the strongest explanatory power (*r*^2^ = 0.629, *P* < 0.001), negatively correlating with RDA1 (−0.82) and positively with RDA2 (0.57). Growth velocity significantly influenced microbial variation (*r*^2^ = 0.478, *P* < 0.001), with negative loadings on both RDA axes (−0.56 for RDA1, −0.83 for RDA2). Birth weight negatively correlated with RDA1 (*r*^2^ = 0.181, *P* = 0.004). Non-significant factors were BMI (*P* = 0.074) and IGF1 (*P* = 0.012). PERMANOVA indicated environmental factors explained 9.0% of total variance (*F* = 1.07, *P* = 0.219), while grouping variables (e.g., treated/untreated groups) significantly impacted community structure (*F* = 1.24, *P* = 0.003), accounting for 6.2% variance. Microbial taxa displayed axis-specific correlations: *g_Lactococcus_A_343306* (0.438) and *g_Fenollaria* (0.338) positively associated with RDA1, while *g_Eubacterium_F* (−0.186) and *g_Amedibacterium* (−0.283) negatively correlated. The study highlights parental height and growth velocity as key drivers, with group-level differences indicating treatment-specific microbial responses.

To assess the relationship between fecal SCFA concentrations and rhGH treatment efficacy for short stature, we performed a RDA (Figure 9B) with age at treatment onset, IGF-1, insulin, birth weight, BMI, and post-treatment growth velocity as explanatory variables, and standardized SCFA levels as the response variable. Results indicate that growth velocity is the sole significant driver of microbial community variation (*r*^2^ = 0.166, *P* = 0.007), while other variables (birth weight, mid-parental height, BMI, IGF-1) were non-significant (*P* > 0.05). SCFAs showed axis-specific correlations: butyric acid (RDA1 = −0.77) and acetic acid (RDA1 = −0.54) negatively correlated with RDA1, aligning with growth velocity effects, whereas hexanoic acid and isovaleric acid weakly positively correlated with RDA2 (loading = 0.20). PERMANOVA confirmed the model's limited explanatory capacity (∼5% variance, *P* = 0.37), with neither environmental factors nor the overall model achieving significance, highlighting unmeasured drivers' dominance.

The study indicates that growth velocity has a moderate impact on microbial communities, with SCFA profiles possibly reflecting growth-related metabolic interactions. However, the model's weak fit highlights the need for further exploration of additional factors or larger sample sizes to better understand the mechanisms driving community assembly. The low explanatory power (<10%) of both models points to significant unmeasured influences, emphasizing the importance of incorporating more variables (e.g., dietary, genetic factors) and using triplot ordination to gain a clearer picture of the complex interplay between the environment, microbes, and host.

## 4 Discussion

### 4.1 Gut microbiota imbalance and functional changes in GHD/ISS children

This study enrolled 61 children with short stature and 41 healthy controls, primarily focusing on the characteristics of fecal microbiota in children with short stature. Children with GHD and ISS may exhibit distinct alterations in their gut microbiota, encompassing changes in microbial composition, metabolic pathways, and functional connectivity. These changes can significantly impact growth, metabolism, and overall health, suggesting potential therapeutic targets for managing these conditions. In our study, children with GHD or ISS displayed gut microbiota dysbiosis, characterized by a decrease in beneficial bacteria such as *Faecalibacterium* and *Akkermansia*, and an increase in potential pathogenic bacteria like *Streptococcus* and *Collinsella* (Figure 3B).

The detailed effects of gut microbiota dysbiosis on GHD and ISS in children involve complex interactions between the microbiome and various physiological processes. *Faecalibacterium*, an anti-inflammatory commensal bacterium, plays a crucial role in maintaining gut homeostasis and immune function. Dysbiosis involving a reduction in *Faecalibacterium* has been associated with increased inflammation and impaired immune regulation, contributing to conditions like inflammatory bowel disease (34). In the context of GHD and ISS, reduced *Faecalibacterium* levels may lead to chronic inflammation, negatively impacting growth and development. *Akkermansia*, known for its ability to degrade mucins and produce SCFAs, is vital for gut health and metabolic processes (35). Dysbiosis involving a decrease in *Akkermansia* has been linked to altered metabolic profiles and an increased risk of metabolic disorders (35). In children with GHD or ISS, reduced *Akkermansia* levels could disrupt metabolic pathways that support growth and development.

*Streptococcus* species are commonly found in the human gut microbiome. While some strains are beneficial, others can be pathogenic. Dysbiosis involving an imbalance of *Streptococcus* species can lead to increased inflammation and oxidative stress, negatively impacting growth and development (36). Specifically, certain *Streptococcus* species have been associated with metabolic disturbances and altered gut barrier function, contributing to conditions like obesity and related growth disorders (36). *Collinsella,* a genus of bacteria typically present in the human gut, has been linked to altered metabolic profiles and an increased risk of metabolic disorders when its levels decrease (35). In children with GHD or ISS, reduced *Collinsella* levels could disrupt metabolic pathways that support growth and development, potentially leading to impaired bone health and muscle mass. Overall, gut microbiota dysbiosis can significantly affect growth and development in children with GHD and ISS, highlighting the importance of maintaining a balanced gut microbiome to support optimal growth and development.

In our study, functional predictions of the microbiota indicated enhanced detoxification metabolic pathways (e.g., atrazine degradation) in the GHD/ISS group, while nutrient-related pathways (e.g., carotenoid synthesis) were impaired. Carotenoids, with their specific structures, can influence cellular processes related to bone development. Evidence suggests that vitamin A (retinol) and carotenoids like beta-carotene are important for bone health, with a study (37) finding positive associations between plasma retinol and dietary intakes of vitamin A and beta-carotene with bone mineral content and density in children aged 6–9 years.

In summary, our present study highlights the significant impact of gut microbiota dysbiosis on the growth and development of children with GHD and ISS, emphasizing the roles of specific bacterial genera and functional pathways. The findings suggest that maintaining a balanced gut microbiome could be a crucial factor in supporting optimal growth and development in these children, potentially opening avenues for new therapeutic approaches.

### 4.2 rhGH treatment-induced fecal microbiota and metabolic pathway changes

After rhGH treatment, we observed an increase in *Bifidobacterium* and butyrate-producing bacteria (e.g., *Faecalibacterium*) and a reduction in pathogenic bacteria like *Collinsella* (Figure 5A). These changes may involve cross-feeding interactions and metabolic shifts. *Bifidobacterium* produces acetate and lactate during carbohydrate fermentation, which are utilized by butyrate-producing bacteria (*Faecalibacterium, Roseburia spp*.) to generate butyrate, enhancing gut homeostasis and suppressing inflammation (38, 39). Lactic acid produced by *Collinsella* can lead to insulin resistance by inhibiting muscle glycolysis and interfering with insulin signaling (40). It can also decrease tight junction protein expression, increase intestinal permeability, and promote lipopolysaccharide entry into the bloodstream, triggering chronic inflammation and further exacerbating insulin resistance (41). A diet with insufficient fiber is linked to higher levels of *Collinsella*, while a high-fiber diet enhances insulin sensitivity by supporting SCFA-producing bacteria (42). *Collinsella* decreases are linked to increased beneficial bacteria. Some bacteria (e.g., *Faecalibacterium, Roseburi*a) produce SCFAs. Butyrate strengthens the intestinal barrier and exhibits anti-inflammatory properties (43), which could indirectly suppress pathogens like *Collinsella*. In summary, increases in *Bifidobacterium* and decreases in *Collinsella* are closely associated with improved host metabolism through mechanisms involving regulation of cholesterol metabolism, repair of insulin signaling pathways, and suppression of inflammation.

Metabolic pathway analysis indicates that rhGH regulates amino acid (glycine-serine metabolism) and lipid metabolism (Figure 6). Metagenomic analyses (44) showed that pathways linked to branched-chain amino acid (BCAA) metabolism were enriched in the gut microbiota of patients with obesity and metabolic syndrome. rhGH may improve insulin resistance by regulating these pathways and reducing BCAAs (45). SCFAs produced by colonic metabolism may enhance protein synthesis by activating the AMPK pathway (14), and rhGH may synergize with this process through the IGF-1 pathway. The gut microbiome influences host lipid metabolism through bile acid metabolism, SCFA synthesis, and other pathways. For example, changes in the Firmicutes/Bacteroidetes ratio correlate with lipid absorption efficiency (46). rhGH may affect these pathways by modulating the gut microbiome.

### 4.3 Association between SCFAs and rhGH treatment efficacy

Our results revealed an association between SCFAs and rhGH treatment efficacy. Acetate and butyrate levels are positively correlated with growth velocity (Figure 7C). In the high efficacy group (growth velocity >97th percentile), the microbiota is dominated by *Lactobacillus* and *Veillonella*, and the levels of SCFAs are higher (Figure 8). Acetic and butyric acid levels have been linked to growth rate, possibly through two mechanisms (47, 48): SCFAs enter the liver via the portal vein, promoting IGF1 synthesis and release; butyric acid activates the growth hormone receptor signaling pathway by increasing glucose uptake in skeletal muscle via the AMPK pathway. rhGH replacement therapy has been shown to enhance intestinal permeability and augment the proliferation of probiotics, facilitating the fermentation of dietary fiber and the generation of SCFAs (49). GH treatment has been observed to reduce visceral fat and increase lean body mass, while also reducing the inhibition of SCFA synthesis by pro-inflammatory factors (50). Lactobacillus produces lactic acid, which *Veillonella* converts to acetic acid in a metabolic network (51).

The present study identified that the fecal microbiota of the high efficacy group was predominantly composed of *Lactobacillus* and *Veillonella*, which may facilitate growth through the following mechanisms: *Lactobacillus* directly ferments dietary fiber to generate acetic and lactic acid (52). It has been observed to suppress chronic inflammation and reduce GH resistance by increasing IL-10 secretion (53). *Veillonella* can convert lactic acid from *Lactobacillus* into propionic and acetic acid, boosting total SCFAs. Propionic acid also indirectly increases glucagon-like peptide-1 (GLP-1) secretion from intestinal L cells, promoting ghrelin secretion (53).

The present study shows how SCFAs like acetic and butyric acids enhance growth velocity by regulating energy metabolism and the inflammatory environment. This process is strengthened by *Lactobacillus* and *Veillonella*, providing a theoretical basis for using intestinal microbiota as a target for complementary therapeutic strategies.

### 4.4 Exploring the gut microbiota—SCFA—GH axis: potential pathways

In our research, GHD/ISS children exhibit gut dysbiosis with reduced beneficial bacteria and increased opportunistic pathogens. rhGH treatment boosts beneficial microbes and curbs potential pathogens, adjusting metabolic pathways. SCFA levels, particularly acetate and butyrate, positively link to growth velocity, being higher in the high-efficacy group. The present findings confirm an association between the gut microbiota, SCFA, and rhGH efficacy; however, the specific causation remains to be elucidated. The potential mechanisms of the gut microbiota-SCFA-GH axis are not yet fully understood. The gut microbiota and host metabolism interact in complex ways, with SCFAs (important metabolic products of the gut microbiota) playing a key role in the microbiota-SCFA-GH axis. Acetate activates FFA2/3 receptors, stimulating intestinal L-cells to secrete GLP-1. This enhances insulin sensitivity and indirectly promotes IGF-1 synthesis, a process vital for regulating host metabolism (54). Butyrate, on the other hand, inhibits HDAC activity to regulate Treg cell differentiation, promoting bone growth and further demonstrating the diverse functions of SCFAs in host physiology (55).

The interaction between the gut microbiota and GH signaling is also significant. *Lactobacillus* (56), through its lactic acid metabolism, lowers intestinal pH, effectively inhibiting pathogen colonization and improving nutrient absorption. *Veillonella* (13) converts lactic acid into propionic acid, activating the mTORC1 pathway. This not only enhances GH receptor sensitivity but also further promotes the host's response to GH signaling.

Moreover, rhGH may indirectly reshape the composition and function of the gut microbiota by increasing intestinal permeability or altering the host's metabolic environment, such as through an increase in IGF-1 levels. Although this hypothesis requires further validation, it offers a new perspective for understanding the interaction between GH signaling and the gut microbiota. These findings not only reveal the complex relationship between the gut microbiota and host metabolism but also provide an important theoretical basis for future research and potential clinical applications.

### 4.5 Comparison with existing literature and points of contradiction

Li et al. (17) observed reduced gut microbiota diversity in children with ISS, but our study revealed no significant differences in *α*-diversity. This discrepancy may stem from variations in sequencing regions (V3–V4 vs. V4) or population characteristics (Eastern Chinese vs. other populations). Magne et al. (57) discussed the Firmicutes/Bacteroidetes ratio in obesity, noting discrepancies in studies may be due to methodological and lifestyle differences. This supports the idea that technical or population differences could lead to different results. Schwarzer et al. (56) demonstrated that *Lactobacillus* supplementation enhances growth in malnutrition models, aligning with the fecal microbiota profile of the high-response subgroup in our study. Evidence suggested SCFAs as key metabolites from gut microbiota, which are produced by fermentation (58). Since Lactobacillus is involved in fermentation, this could be a connection. However, Huang et al. (15) reported *Bacteroidetes* dominance in the gut microbiota of GHD children, whereas our findings indicated *Firmicutes* predominance. This divergence may reflect differences in dietary controls (e.g., exclusion of vegetarians) or age ranges. Diet can affect the Firmicutes/Bacteroidetes ratio; studies (59) show that a high-fat diet increases Firmicutes and decreases Bacteroidetes in mice, but human studies may vary. Additionally, while Yan et al. (13) proposed that gut microbiota directly stimulates IGF-1 via SCFAs, our study detected limited SCFA alterations, suggesting potential host metabolic compensation or insufficient treatment duration (2 years) to elicit measurable changes.

## 5 Study limitations

The present study is subject to several key methodological limitations. First, the relatively small sample size (*n* = 61), particularly in the subgroup with the highest efficacy (>97th percentile), may reduce statistical power and introduce bias in the assessment of metabolic heterogeneity (e.g., the difference in butyrate did not reach statistical significance). Second, although the study population included vegetarians were excluded, confounding factors such as dietary fiber intake and physical activity were not accurately quantified, which, coupled with the fact that the dietary homogeneity may interfere with the analysis of intestinal microbiome; third, the prediction of intestinal microbiota function relied only on the PICRUSt2 algorithm, lacked macro-genome sequencing to verify the accuracy of metabolic pathways, and did not directly detect key biomarkers, such as plasma SCFA concentration, through metabolomics. Finally, the study design did not incorporate an animal model or fecal colony transplantation experiments, which did not allow for the verification of causal mechanisms between the intervention of rhGH and the modulation of the microbiota.The study design did not include animal models or fecal colony transplantation experiments. In future studies, it is imperative to augment the sample size, analyze biomarkers across multiple groups, and validate the mechanism through experimental models to enhance the depth and reliability of the study.

## 6 Future research directions

In terms of clinical translation, randomized controlled trials should be designed to combine rhGH with probiotics (such as *Bifidobacterium* and *Lactobacillus*) to evaluate their synergistic therapeutic effects. The feasibility of using SCFAs supplements (such as butyrate) as an adjunct to rhGH treatment should also be explored. To deepen the understanding of the mechanisms involved, an integrated multi—omics approach combining metagenomics, metabolomics, and host transcriptomics should be employed to unravel the specific pathways linking the gut microbiota, metabolites, and GH signaling (such as the cross—regulation between mTORC1 and FFA3). Animal models, such as GHD mice, should be established to verify the enhancing effect of specific gut microbiota (such as *Veillonella*) on the efficacy of rhGH through fecal microbiota transplantation. In terms of population expansion, multicenter cohort studies should be conducted to include children from different regions and dietary cultures (such as eastern vs. western), with the variable of dietary fiber intake controlled to reduce bias. Long—term follow—up studies should also be carried out, extending the treatment duration to 5 years to observe the sustained changes in the gut microbiota and SCFAs and their association with adult height.

## 7 Conclusion

This study, for the first time, reveals that rhGH treatment is associated with improved growth rate of children with short stature, along with modulations in the gut microbiota (increasing beneficial bacteria and reducing pathogenic bacteria) and certain SCFAs (such as acetate). However, the complete mechanism of the gut microbiota—SCFA—GH axis still needs further validation. In the future, combining interventional studies with multi—omics technologies may lead to the development of a “microbiota—hormone” combination therapy, which could optimize treatment strategies for children with growth disorders.

## Data Availability

The datasets presented in this study can be found in online repositories. The names of the repository/repositories and accession number(s) can be found in the article/Supplementary Material.
