# Supplementary material for: Gut microbiome and short-chain fatty acids associated with the efficacy of growth hormone treatment in children with short stature
Source: Front Pediatr. 2025 Mar 31;13:1557878. doi: 10.3389/fped.2025.1557878 (PMC11994682; doi:10.3389/fped.2025.1557878)
Supplement: Supplementary file 1 [file Supplementaryfile1.docx]

Supplementary Table S1. Reference Values for Annual Growth Rate Charts (cm/y) for Girls and Boys.

| *Age years* |  | Girls | | | | |  |  | Boys | | | | |  |
| --- | --- | --- | --- | --- | --- | --- | --- | --- | --- | --- | --- | --- | --- | --- |
|  |  | *Percentile* | | | | |  |  | *Percentile* | | | | |  |
|  | 3^rd^ | 10^th^ | 25^th^ | 50^th^ | 75^th^ | 90^th^ | 97^th^ | 3^rd^ | 10^th^ | 25^th^ | 50^th^ | 75^th^ | 90^th^ | 97^th^ |
| 6 | 3.84 | 4.56 | 5.3 | 6.1 | 6.9 | 7.64 | 8.36 | 3.84 | 4.56 | 5.29 | 6.1 | 6.91 | 7.64 | 8.36 |
| 7 | 3.47 | 3.99 | 4.59 | 5.22 | 5.85 | 6.45 | 6.97 | 3.8 | 4.17 | 4.85 | 5.32 | 5.79 | 6.47 | 6.84 |
| 8 | 3.85 | 4.55 | 5.25 | 6.03 | 6.81 | 7.51 | 8.21 | 3.6 | 4.1 | 5.05 | 5.7 | 6.35 | 7.3 | 7.8 |
| 9 | 2.77 | 3.54 | 4.31 | 5.17 | 6.05 | 6.82 | 7.59 | 3.12 | 3.63 | 4.15 | 4.72 | 5.29 | 5.81 | 6.32 |
| 10 | 3.77 | 4.93 | 5.91 | 7.07 | 8.23 | 9.21 | 10.37 | 4.25 | 4.74 | 5.24 | 5.78 | 6.32 | 6.82 | 7.3 |
| 11 | 3.53 | 4.46 | 5.41 | 6.46 | 7.51 | 8.46 | 9.39 | 2.47 | 3.46 | 4.44 | 5.53 | 6.62 | 7.6 | 8.59 |
| 12 | 1.87 | 3.29 | 4.72 | 6.31 | 7.9 | 9.33 | 10.75 | 2.96 | 4.77 | 6.59 | 8.62 | 10.65 | 12.47 | 14.28 |

This table is based on relevant research reports from South Korea(1) and Hong Kong, China(2).

Supplementary Table S2. High-abundance or group-characteristic genera and their full taxonomic classifications

| Species | Full Taxonomic Path |
| --- | --- |
| *g_Bifidobacterium_388775* | d_Bacteria;p_Actinobacteriota;c_Actinomycetia;o_Actinomycetales;f_Bifidobacteriaceae;g_Bifidobacterium_388775 |
| *g_Thermophilibacter* | d_Bacteria;p_Actinobacteriota;c_Coriobacteriia;o_Coriobacteriales;f_Atopobiaceae;g_Thermophilibacter |
| *g_Desulfovibrio_R_446353* | d_Bacteria;p_Desulfobacterota_I;c_Desulfovibrionia;o_Desulfovibrionales;f_Desulfovibrionaceae;g_Desulfovibrio_R_446353 |
| *g_Blautia_A_141781* | d_Bacteria;p_Firmicutes_A;c_Clostridia_258483;o_Lachnospirales;f_Lachnospiraceae;g_Blautia_A_141781 |
| *g_Roseburia* | d_Bacteria;p_Firmicutes_A;c_Clostridia_258483;o_Lachnospirales;f_Lachnospiraceae;g_Roseburia |
| *g_Emergencia* | d_Bacteria;p_Firmicutes_A;c_Clostridia_258483;o_Peptostreptococcales;f_Anaerovoracaceae;g_Emergencia |
| *g_CAG_269* | d_Bacteria;p_Firmicutes_A;c_Clostridia_258483;o_TANB77;f_CAG_508;g_CAG_269 |
| *g_CAG_41* | d_Bacteria;p_Firmicutes_A;c_Clostridia_258483;o_UBA1381;f_UBA1381;g_CAG_41 |
| *g_Enterococcus_B* | d_Bacteria;p_Firmicutes_D;c_Bacilli;o_Lactobacillales;f_Enterococcaceae;g_Enterococcus_B |
| *g_Enterococcus_H_360604* | d_Bacteria;p_Firmicutes_D;c_Bacilli;o_Lactobacillales;f_Enterococcaceae;g_Enterococcus_H_360604 |
| *g_Lacticaseibacillus* | d_Bacteria;p_Firmicutes_D;c_Bacilli;o_Lactobacillales;f_Lactobacillaceae;g_Lacticaseibacillus |
| *g_Leuconostoc_B* | d_Bacteria;p_Firmicutes_D;c_Bacilli;o_Lactobacillales;f_Lactobacillaceae;g_Leuconostoc_B |
| *g_RUG13038* | d_Bacteria;p_Firmicutes_D;c_Bacilli;o_RF39;f_UBA660;g_RUG13038 |
| *g_Staphylococcus* | d_Bacteria;p_Firmicutes_D;c_Bacilli;o_Staphylococcales;f_Staphylococcaceae;g_Staphylococcus |
| *g_Cetobacterium_A* | d_Bacteria;p_Fusobacteriota;c_Fusobacteriia;o_Fusobacteriales_993521;f_Fusobacteriaceae_993521;g_Cetobacterium_A |
| *g_Ralstonia* | d_Bacteria;p_Proteobacteria;c_Gammaproteobacteria;o_Burkholderiales_592524;f_Burkholderiaceae_A_580492;g_Ralstonia |
| *g_Klebsiella_724518* | d_Bacteria;p_Proteobacteria;c_Gammaproteobacteria;o_Enterobacterales_A_737866;f_Enterobacteriaceae_A;g_Klebsiella_724518 |
| *g_Pseudomonas_E_647464* | d_Bacteria;p_Proteobacteria;c_Gammaproteobacteria;o_Pseudomonadales_650611;f_Pseudomonadaceae;g_Pseudomonas_E_647464 |
| *g_Pyramidobacter* | d_Bacteria;p_Synergistota;c_Synergistia;o_Synergistales;f_Dethiosulfovibrionaceae;g_Pyramidobacter |
| *g_Akkermansia* | d_Bacteria;p_Verrucomicrobiota;c_Verrucomicrobiae;o_Verrucomicrobiales;f_Akkermansiaceae;g_Akkermansia |
| *g_Collinsella* | d_Bacteria;p_Actinobacteriota;c_Coriobacteriia;o_Coriobacteriales;f_Coriobacteriaceae;g_Collinsella |
| *g_Bacteroides_H* | d_Bacteria;p_Bacteroidota;c_Bacteroidia;o_Bacteroidales;f_Bacteroidaceae;g_Bacteroides_H |
| *g_Phocaeicola_A_858004* | d_Bacteria;p_Bacteroidota;c_Bacteroidia;o_Bacteroidales;f_Bacteroidaceae;g_Phocaeicola_A_858004 |
| *g_Clostridium_T* | d_Bacteria;p_Firmicutes_A;c_Clostridia_258483;o_Clostridiales;f_Clostridiaceae_222000;g_Clostridium_T |
| *g_Agathobacter_164117* | d_Bacteria;p_Firmicutes_A;c_Clostridia_258483;o_Lachnospirales;f_Lachnospiraceae;g_Agathobacter_164117 |
| *g_Anaerobutyricum* | d_Bacteria;p_Firmicutes_A;c_Clostridia_258483;o_Lachnospirales;f_Lachnospiraceae;g_Anaerobutyricum |
| *g_Anaerostipes* | d_Bacteria;p_Firmicutes_A;c_Clostridia_258483;o_Lachnospirales;f_Lachnospiraceae;g_Anaerostipes |
| *g_Dorea_A* | d_Bacteria;p_Firmicutes_A;c_Clostridia_258483;o_Lachnospirales;f_Lachnospiraceae;g_Dorea_A |
| *g_Fusicatenibacter* | d_Bacteria;p_Firmicutes_A;c_Clostridia_258483;o_Lachnospirales;f_Lachnospiraceae;g_Fusicatenibacter |
| *g_Mediterraneibacter_A_155507* | d_Bacteria;p_Firmicutes_A;c_Clostridia_258483;o_Lachnospirales;f_Lachnospiraceae;g_Mediterraneibacter_A_155507 |
| *g_Ruminococcus_B* | d_Bacteria;p_Firmicutes_A;c_Clostridia_258483;o_Lachnospirales;f_Lachnospiraceae;g_Ruminococcus_B |
| *g_Ruminococcus_E* | d_Bacteria;p_Firmicutes_A;c_Clostridia_258483;o_Oscillospirales;f_Acutalibacteraceae;g_Ruminococcus_E |
| *g_Faecalibacterium* | d_Bacteria;p_Firmicutes_A;c_Clostridia_258483;o_Oscillospirales;f_Ruminococcaceae;g_Faecalibacterium |
| *g_Gemmiger_A_73129* | d_Bacteria;p_Firmicutes_A;c_Clostridia_258483;o_Oscillospirales;f_Ruminococcaceae;g_Gemmiger_A_73129 |
| *g_CAG-41* | d_Bacteria;p_Firmicutes_A;c_Clostridia_258483;o_UBA1381;f_UBA1381;g_CAG-41 |
| *g_Faecalibacillus* | d_Bacteria;p_Firmicutes_D;c_Bacilli;o_Erysipelotrichales;f_Coprobacillaceae;g_Faecalibacillus |
| *g_Streptococcus* | d_Bacteria;p_Firmicutes_D;c_Bacilli;o_Lactobacillales;f_Streptococcaceae;g_Streptococcus |

### Supplementary Table S3. Fecal short chain fatty acid concentrations in children with short stature

| Acid Type | Group | n | Minimum | 25th Percentile | Median | 75th Percentile | Maximum |
| --- | --- | --- | --- | --- | --- | --- | --- |
|  |  |  | Concentration μg/g | | | | |
| Acetic Acid | GHD_untreated | 16 | 173.6 | 578.3 | 994.6 | 1573 | 3429 |
|  | GHD_treated | 17 | 121.4 | 1395 | 1904 | 2394 | 4515 |
|  | ISS_untreated | 16 | 482.3 | 1175 | 1496 | 2689 | 4454 |
|  | ISS_treated | 12 | 106.9 | 920.7 | 1702 | 2344 | 3229 |
| Butyric Acid | GHD_untreated | 16 | 70.53 | 233.5 | 426.7 | 574.2 | 889.9 |
|  | GHD_treated | 17 | 28.42 | 266.7 | 633.9 | 991.5 | 1713 |
|  | ISS_untreated | 16 | 115.4 | 325.1 | 561 | 1170 | 1922 |
|  | ISS_treated | 12 | 42.05 | 201.3 | 427.2 | 916.1 | 1507 |
| Hexanoic Acid | GHD_untreated | 16 | 3.377 | 3.822 | 5.567 | 9.365 | 26.54 |
|  | GHD_treated | 17 | 2.899 | 3.771 | 5.167 | 10.12 | 43.13 |
|  | ISS_untreated | 16 | 3.102 | 3.924 | 7.037 | 7.451 | 95.31 |
|  | ISS_treated | 12 | 3.231 | 4.301 | 7.204 | 11.62 | 91.55 |
| Isobutyric Acid | GHD_untreated | 16 | 19.06 | 31.91 | 59.71 | 92.37 | 245.3 |
|  | GHD_treated | 17 | 8.731 | 21.8 | 85.24 | 174 | 345.9 |
|  | ISS_untreated | 16 | 13.67 | 46.23 | 82.2 | 124.2 | 198.2 |
|  | ISS_treated | 12 | 57.11 | 70.17 | 106.6 | 156.3 | 262.1 |
| Isovaleric Acid | GHD_untreated | 16 | 16.01 | 17.81 | 53.8 | 96.26 | 265.7 |
|  | GHD_treated | 17 | 9.463 | 19.87 | 68.54 | 165.8 | 376.4 |
|  | ISS_untreated | 16 | 13.06 | 30.1 | 74.85 | 124.3 | 192.7 |
|  | ISS_treated | 12 | 34.67 | 59.09 | 110.2 | 149.4 | 296.6 |
| Propionic Acid | GHD_untreated | 16 | 177.2 | 356.9 | 428.7 | 839.6 | 1624 |
|  | GHD_treated | 17 | 27.11 | 368 | 682.2 | 950.5 | 1376 |
|  | ISS_untreated | 16 | 70.42 | 504.4 | 758.5 | 979.8 | 2242 |
|  | ISS_treated | 12 | 265 | 524.2 | 617.4 | 757.9 | 1347 |
| Valeric Acid | GHD_untreated | 16 | 16.05 | 26.71 | 58.92 | 137.9 | 275.9 |
|  | GHD_treated | 17 | 11.46 | 23.37 | 52.92 | 175.3 | 343.1 |
|  | ISS_untreated | 16 | 11.78 | 37.26 | 126.8 | 191.1 | 564.5 |
|  | ISS_treated | 12 | 27.81 | 89.85 | 139.3 | 205 | 286 |


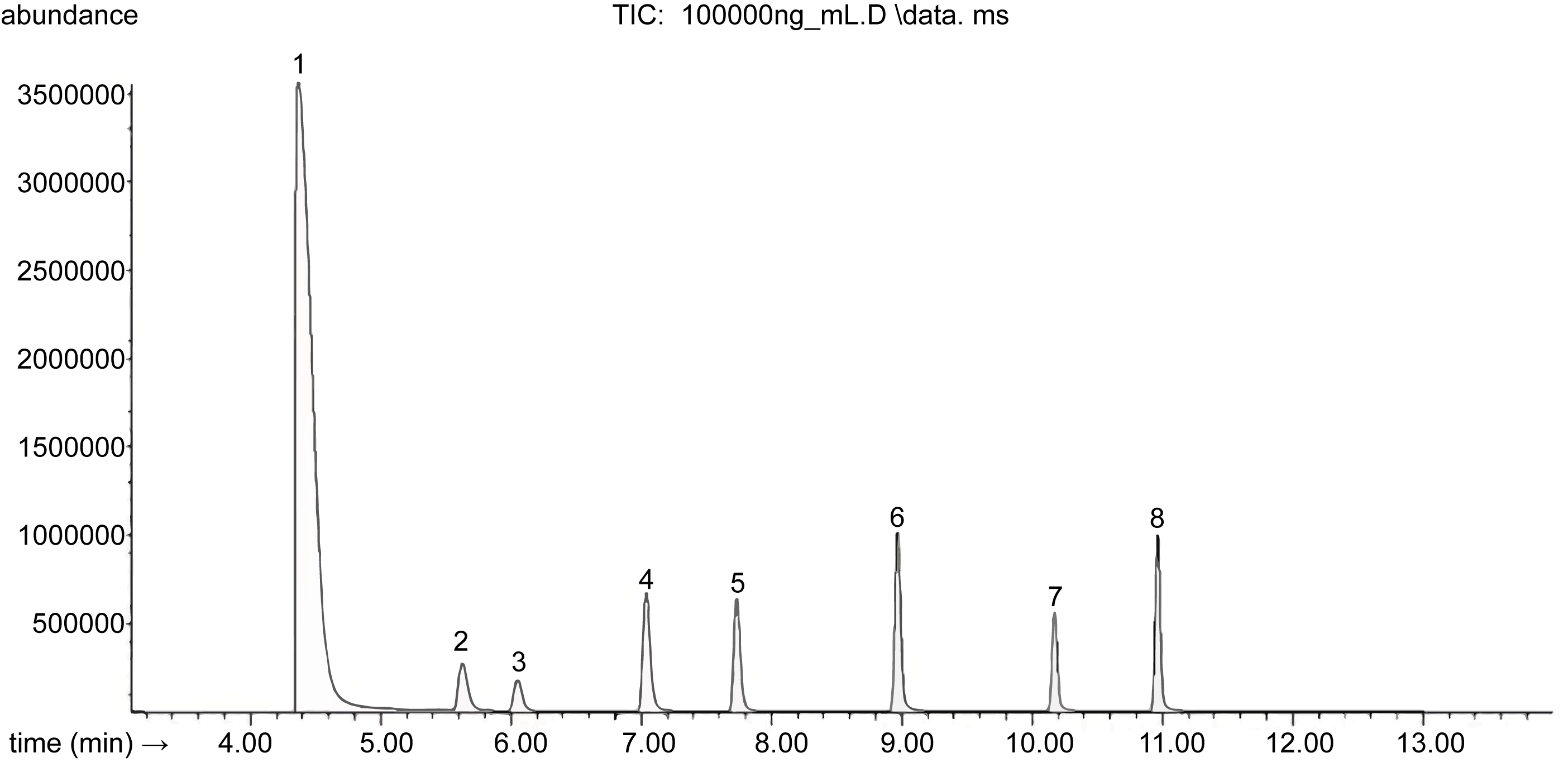


Supplementary Figure S1. The TIC chromatogram of mixed reference SCFAs. 1. acetic acid, 2. propionic acid, 3. Isobutyric acid, 4. Butyric acid, 5. Isovaleric acid, 6. Valeric acid, 7. 4-methylvaleric acid, 8. Hexanoic acid. A mixed standard solution with a concentration of 50 μg/mL for each standard was continuously injected six times on the GC-MS, and the relative standard deviation (RSD) was calculated based on the peak area ratio of each component to the internal standard.

**References:**

1.Ghaemmaghami P, Ayatollahi S, Alinejad V, Haem E. Longitudinal standards for growth velocity of infants from birth to 4 years born in West Azerbaijan Province of northwest Iran. *EPIDEMIOL HEALTH*.(2015)37: e2015029. doi:10.4178/epih/e2015029

2.Liang SF, Dai WZ. A growth velocity standard for Chinese children in Hong Kong. *CHINESE MED J-PEKING*.(1989)102: 233-5.
